# Baicalein resensitizes tamoxifen‐resistant breast cancer cells by reducing aerobic glycolysis and reversing mitochondrial dysfunction via inhibition of hypoxia‐inducible factor‐1α

**DOI:** 10.1002/ctm2.577

**Published:** 2021-11-04

**Authors:** Yan Chen, Jingyu Zhang, Minqin Zhang, Yuxuan Song, Yue Zhang, Shuangqin Fan, Shuang Ren, Lingyun Fu, Nenling Zhang, Hui Hui, Xiangchun Shen

**Affiliations:** ^1^ The State Key Laboratory of Functions and Applications of Medicinal Plants Guizhou Medical University Guizhou China; ^2^ The High Efficacy Application of Natural Medicinal Resources Engineering Center of Guizhou Province School of Pharmaceutical Sciences Guizhou Medical University Guizhou China; ^3^ The Union Key Laboratory of Guiyang City‐Guizhou Medical University School of Pharmaceutical Sciences Guizhou Medical University Guizhou China; ^4^ The Key Laboratory of Optimal Utilization of Natural Medicine Resources School of Pharmaceutical Sciences Guizhou Medical University Guizhou China; ^5^ State Key Laboratory of Natural Medicines Jiangsu Key Laboratory of Carcinogenesis and Intervention China Pharmaceutical University Nanjing China

**Keywords:** aerobic glycolysis, baicalein, hypoxia‐inducible factor‐1α, mitochondrial dysfunction, resistance, tamoxifen

## Abstract

Drug resistance is a major hurdle for the effectiveness of tamoxifen (TAM) to provide clinical benefit. Therefore, it is essential to identify a sensitizer that could be used to improve TAM efficacy in treating TAM‐resistant breast cancer. Here, we investigated the ability of baicalein to reverse TAM resistance. We found that baicalein increased the efficacy of TAM in inhibiting proliferation and inducing apoptosis of TAM‐resistant cells. It also enhanced the TAM‐induced growth reduction of resistant cells from NOD/SCID mouse mammary fat pads, without causing obvious systemic toxicity. Analyses using the CellMiner tool and the Kaplan–Meier plotter database showed that HIF‐1α expression was inversely correlated with TAM therapeutic response in NCI‐60 cancer cells and breast cancer patients. HIF‐1α expression was increased in TAM‐resistant cells due to an increase in mRNA levels and reduced ubiquitin‐mediated degradation. Baicalein reduced HIF‐1α expression by promoting its interaction with PHD2 and pVHL, thus facilitating ubiquitin ligase‐mediated proteasomal degradation and thereby suppressing the nuclear translocation, binding to the hypoxia‐response element, and transcriptional activity of HIF‐1α. As a result, baicalein downregulated aerobic glycolysis by restricting glucose uptake, lactate production, ATP generation, lactate/pyruvate ratio and expression of HIF‐1α‐targeted glycolytic genes, thereby enhancing the antiproliferative efficacy of TAM. Furthermore, baicalein interfered with HIF‐1α inhibition of mitochondrial biosynthesis, which increased mitochondrial DNA content and mitochondrial numbers, restored the generation of reactive oxygen species in mitochondria, and thus enhanced the TAM‐induced mitochondrial apoptotic pathway. The HIF‐1α stabilizer dimethyloxallyl glycine prevented the baicalein‐induced downregulation of glycolysis and mitochondrial biosynthesis and reduced the effects of baicalein on reversing TAM resistance. Our results indicate that baicalein is a promising candidate to help overcome TAM resistance by sensitizing resistant cells to TAM‐induced growth inhibition and apoptosis. The mechanism underlying the effects of baicalein consists of inhibition of HIF‐1α–mediated aerobic glycolysis and mitochondrial dysfunction.

## Graphical abstract

• Baicalein possesses the ability to reverse TAM resistance.

• Baicalein reduces the expression and activity of HIF‐1α via the promotion of its interaction with PHD2 and pVHL and facilitates the ubiquitin ligase‐mediated proteasomal degradation in TAM‐resistant cells.

• Baicalein inhibits HIF‐1α–mediated aerobic glycolysis and relieves HIF‐1α–restrained mitochondrial biosynthesis and reactive oxygen species generation, thereby enhancing TAM‐induced proliferation and mitochondrial‐mediated apoptosis.

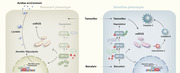

## 1 INTRODUCTION

Breast cancer is the most common cancer and the leading cause of death in women worldwide,^1^ with the oestrogen receptor (ER)–positive subtype accounting for more than 70% of all diagnosed breast cancers.^2^ The growth and development of ER‐positive breast cancer is thought to be oestrogen hormone dependent, and thus endocrine agents that abrogate oestrogenic signalling or oestrogen production represent cornerstone pharmacological therapies for the treatment of this subtype.^3^ Tamoxifen (TAM) is a selective ER modulator and the most widely prescribed endocrine agent used for both pre‐ and postmenopausal women.^4^ Although TAM represents a tremendous therapeutic breakthrough with great improvements in survival, not all patients benefit from its use.^5^ Approximately 25% of patients do not respond to initial TAM treatment, and 40% of the responsive patients develop resistance.^5^ This emergence of intrinsic and acquired resistance represents a substantial obstacle to patient life expectancy.^6^ Therefore, exploration of new therapeutic strategies to enhance the efficacy of TAM by overcoming resistance in breast cancer cells is urgently needed.

Previous investigations have proposed that hypoxia‐inducible factor‐1α (HIF‐1α) is a potential druggable target for resistance treatment during anticancer therapy.^7^ Analysis of clinical samples has revealed that high expression levels of HIF‐1α do not only predict poor prognosis for ER‐positive breast cancer patients, but are also positively associated with resistance to primary endocrine therapy.^8^ In general, HIF‐1α is hydroxylated by prolyl hydroxylase (PHD), which leads to its proteasomal degradation via E3 ubiquitin ligase under normoxia.^9^ Proline hydroxylation of HIF‐1α is suppressed during hypoxia, and the stabilized HIF‐1α can translocate into the nucleus, interacts with HIF‐1β and binds to the hypoxia‐response element (HRE) to transcriptionally activate multiple genes.^10^ However, HIF‐1α expression is upregulated, and transcriptional activity is activated in TAM‐resistant human breast cancer cell lines, even under normoxic conditions.^11^ Pharmacological inhibition or knockdown of HIF‐1α expression sensitizes resistant breast cancer cells to TAM and other endocrine‐therapy agents,^8^, ^11^ which suggests that HIF‐1α may be a promising target for the treatment of TAM‐resistant breast cancer.

It has been shown that in addition to its inhibition of ERα signalling, TAM activates the mitochondria‐mediated apoptotic pathway.^12^ Nevertheless, TAM also behaves as a mitochondrial toxin because it can act as both an uncoupling agent and a potent inhibitor of the electron transport chain and decrease the effects of complex III and IV, leading to mitochondrial failure.^13^, ^14^ More specifically, TAM treatment also has a direct deleterious effect on mitochondrial DNA (mtDNA) content and replication.^15^, ^16^ Thus, TAM‐resistant cells exhibit mitochondrial damage, with a reduction in mitochondrial content and impaired ability to induce apoptosis,^15^, ^16^ along with enhanced aerobic glycolysis as their main energy source.^11^, ^17^ The rate of glucose uptake in glycolysis is 100 times faster than that in oxidative phosphorylation (OXPHOS), and this metabolic pathway provides a selective growth advantage for resistant cells whereby they can rapidly generate ATP from conversion of pyruvate to lactate.^18^ HIF‐1α is a central transcription factor controlling the expression of many glycolytic genes,^19^ and it is also responsible for decreasing mitochondrial number and function by inducing mitophagy or inhibiting mitochondrial biogenesis.^20^, ^21^ Thus, it has been suggested that inhibition of HIF‐1α expression or function may represent an attractive therapeutic strategy to decrease aerobic glycolysis and increase mitochondrial biogenesis and apoptotic response, which would increase the efficacy of TAM in resistant cells.

Baicalein is one of the main active ingredients derived from the root of *Scutellaria baicalensis* Georgi, a traditional Chinese herbal medicine, and has extensive anticancer effects. In particular, we and other researchers have found that baicalein has potent antiestrogenic activity and is a promising compound for the treatment of ER‐positive breast cancer.^22^, ^23^, ^24^ Importantly, baicalein and TAM depress estrogenic effects via different mechanisms.^25^ Furthermore, previous studies have also identified baicalein as a sensitizer for anticancer therapy in radio‐ and chemoresistant breast cancer cells.^26^, ^27^ Baicalein can inhibit the function of HIF‐1α in several types of cancer cells. For example, it suppresses hypoxia‐induced HIF‐1α accumulation and transcriptional activation in gastric cancer AGS cells^28^ and mTOR signalling‐mediated HIF‐1α activation in melanoma cells.^29^ On the basis of these data, baicalein may represent a potential novel therapy for reversal of TAM resistance in breast cancer.

In the present study, we investigated the ability of baicalein to resensitize TAM‐resistant breast cancer cells both in vitro and in vivo, and explored the potential mechanisms underlying HIF‐1α–mediated aerobic glycolysis and mitochondrial dysfunction.

## 2 MATERIALS AND METHODS

### 2.1 Reagents and antibodies

Details regarding the reagents and antibodies used in this investigation are listed in the Supplementary Information. Baicalein, TAM, 4‐hydroxytamoxifen (OHT), *N*‐acetyl‐L‐cysteine (NAC), and dimethyloxallyl glycine (DMOG) were dissolved in dimethylsulfoxide (DMSO) at 0.1‐0.2 M and stored at −20°C.

### 2.2 Cell lines

The TAM‐resistant cell lines (ATCC, Manassas, VA, USA) included MCF‐7TR (CRL‐3435, MCF‐7 Tam1), T‐47DTR (CRL‐3436, T‐47D Tam1), BT‐474TR (CRL‐3433, BT‐474 Tam1) and ZR‐75‐1TR (CRL‐3438, ZR‐75‐1 Tam1). The parental cell lines (Kunming Cell Bank, Yunnan, China) included MCF‐7, T‐47D, BT‐474 and ZR‐75‐1). The TAM‐resistant cells were cultured according to standard ATCC protocols and were supplemented with 1 μM OHT in the culture medium to maintain the TAM resistance. More details are provided in the Supplementary Information.

### 2.3 Cell proliferation assays

Cell growth was detected using a trypan blue dye exclusion assay and a cell counting system (JIMBIO FIL, Zhuowei Technology, Jiangsu, China). The cells were treated with OHT (1 μM) or co‐treated with various concentrations of baicalein (6.25, 12.5 and 25 μM) for 72 h, and a cell growth curve was plotted using counted cell numbers every 24 h. The 50% inhibitory concentration (IC_50_) of OHT or OHT combined with baicalein was calculated using an MTT assay. After 24‐h incubation in 96‐well plates, cells were treated with various OHT concentrations with or without baicalein (25 μM) for 48 h. After treatment, the cells were incubated with MTT solution (5 mg/ml, 20 μl/well, 4 h), and the medium was then substituted using DMSO (100 μl/well). The detection of optical density (OD) values was performed using a microplate reader set at 570 nm (BioTek, Winooski, VT, USA). Cell viability was calculated by comparing OD values to those for non‐treated cells. Colony‐formation assays were performed using the double‐layer soft‐agar system. Cells (5000 cells/well) were seeded into 0.35% agarose as the upper layer, with 0.7% agarose solidified for the lower layer, in six‐well plates and cultured for 21 days. The indicated treatment medium (1 ml) was then supplemented every 3 days. After the assay, the colonies were detected using 0.1% crystal violet staining and a ChemiDoc XRS^+^ system under white light patterning (Bio‐Rad, Hercules, CA, USA), with results analysed using ImageJ software (v.1.8.0; Bethesda, MD, USA).

### 2.4 Detection of apoptosis

Apoptotic cells were detected using an annexin V/PI staining kit. Detection was performed using a NovoCyte flow cytometer with NovoExpress analysis software (ACEA Biosciences, San Diego, CA, USA).

### 2.5 Western blotting

Total cellular protein was extracted using immunoprecipitation assay lysis buffer containing PMSF (1 mM) and protease inhibitor cocktail (PIC, 1:100). Mitochondrial and nuclear proteins were extracted using a cell mitochondria isolation kit and a nuclear/cytoplasmic protein extraction kit, respectively, both supplemented with PMSF (1 mM) and PIC (1:100). Subsequent procedures were performed as described in a previous investigation.^30^ The proteins were finally detected using a chemiluminescent horseradish peroxidase substrate, and scanned using a ChemiDoc XRS^+^ system with Image Lab Software (v.5.2; Bio‐Rad).

### 2.6 Immunoprecipitation

Immunoprecipitation was performed using a protein A/G plus agarose immunoprecipitation kit and the immunoprecipitates were analysed via western blotting.

### 2.7 Immunofluorescence staining

Cells were incubated on coverslips in six‐well plates. After treatment, the coverslips were fixed in methyl alcohol (10 min, −20°C), permeabilized with 0.1% Triton‐X100 (15 min, 4°C), and blocked with goat serum (40 min, 4°C). The coverslips were then sequentially incubated with HIF‐1α antibody (1:100, overnight, 4°C), FITC‐conjugated secondary antibody (1:500, 1 h, 37°C), and DAPI (1 μg/ml, 30 min, 25°C). Then the cellular localization of HIF‐1α was estimated using a Leica DMi8 fluorescence microscope (Wetzlar, Germany, 400 × magnification) and ImageJ software (v.1.8.0).

### 2.8 Real‐time PCR

Total RNA was harvested using a MiniBEST universal RNA extraction kit. The purity and concentration of the isolated RNA were evaluated using a N60 Touch nanophotometer (Implen, Munich, Germany). The mRNA was then reverse‐transcribed (RT) to produce cDNA using an RT reagent with a gDNA eraser kit in a thermal cycler (SimpliAmp, Applied Biosystems, Carlsbad, CA, USA) and amplified using primers (Table S1; Sangon Biotech, Shanghai, China) and TB Green Premix Ex Taq II reagents in a real‐time PCR detection system (CFX96; Bio‐Rad). Calculation of mRNA levels utilized the 2^−ΔΔCt^ method with *β‐actin* as the endogenous control.

### 2.9 Electrophoretic mobility shift assay (EMSA)

Double‐stranded probes for the HIF‐1α consensus oligonucleotide were purchased from Beyotime (Nanjing, China), including biotinylated native probes (GS032B), biotinylated mutated probes (GS032T), and non‐biotinylated native probes (GS032A). Nuclear protein (5 μg) was mixed with the probes for 30 min at 25°C, and then EMSA was performed using a chemiluminescent EMSA kit and maximum‐sensitivity substrate, with signals visualized using a ChemiDoc XRS^+^ system (Bio‐Rad).

### 2.10 Luciferase reporter assay

Cells were transfected with the HRE‐luciferase reporter plasmid (pGL3‐HRE) and pRL Renilla luciferase control reporter vector plasmid (pRL‐TK vector) using Lipofectamine 2000 reagent at a ratio of 10:1 in six‐well plates. After treatment, the Dual‐Luciferase reporter assay system was used to detect HRE‐luciferase activity, which was normalized to Renilla luciferase activity for quantification.

### 2.11 Glucose uptake assay

Glucose uptake was determined using the fluorescent glucose analogue 2‐nitrobenzodeoxyglucose (2‐NBDG). After treatment, cells were incubated in phenol red‐free and glucose‐free medium for 12 h, followed by 30‐min incubation with 100 μM 2‐NBDG in the same medium. Then glucose uptake was measured using a NovoCyte flow cytometer, and the fluorescence intensity was analysed using NovoExpress software (ACEA Biosciences).

### 2.12 Measurement of L‐lactate content

Extracellular and intracellular L‐lactate levels were evaluated using a colorimetric/fluorometric L‐lactate assay kit. Cells were treated for 48 h in phenol‐free and serum‐free medium. Then the culture medium and cells were collected for lactate measurement. The fluorescence absorbance was measured using a Varioskan multimode microplate spectrophotometer at Ex/Em = 535/587 nm (Thermo Fisher Scientific, Waltham, MA, USA).

### 2.13 Determination of ATP content

The ATP content in cell extracts was measured using an ATP determination kit. Cells were lysed in lysis buffer with PMSF (1 mM) and PIC (1:100). Then ATP levels were measured as the luminescence intensity at 560 nm using a Varioskan microplate spectrophotometer (Thermo), with the ATP content normalized to the cell protein content.

### 2.14 Measurement of lactate and pyruvate levels

Lactate and pyruvate levels were measured using liquid chromatography–tandem mass spectrometry (LC‐MS) as described in the Supplementary Information.

### 2.15 Transmission electron microscopy

Mitochondrial morphology and structure were analysed via transmission electron microscopy (TEM) at 80 kV (HT7800, Hitachi, Tokyo, Japan). After treatment, cells were fixed in 2.5% glutaraldehyde (pH 7.35 phosphate buffer, overnight, 4°C) and incubated in 1% osmium tetroxide for 2 h. Cells were then dehydrated using an ethanol gradient (50%, 70%, 90% and 100%, 15 min for each) and embedded in Epon 812. Ultrathin sections (70 nm) were prepared using an EM UC7 ultramicrotome (Leica, Wetzlar, Germany). These sections were stained with lead citrate and 2% uranyl acetate for 15 min for viewing on the compact digital TEM. The mitochondrial morphology (length divided by width: round: ≤ 1.5; intermediate: 1.5 to 3.0; elongated: > 3, *n* = 6 cells per group) and mitochondrial area (*n* = 30 individual mitochondria per group) were calculated using ImageJ software (v.1.8.0).

### 2.16 mtDNA copy‐number quantification

Total cellular DNA was separated using a MiniBEST universal genomic DNA extraction kit. The mtDNA content was quantified by estimating the relative levels of mtDNA‐*ND1* to *SLCO2B1* and mtDNA‐*ND5* to *SERPINA1* using a TB Green Premix Ex Taq II kit in a CFX96 real‐time PCR detection system (Bio‐Rad). The amplification primers were obtained from a Human Mitochondrial DNA Monitoring Primer Set and the mtDNA copy number was calculated as (2^(^ *^SLCO2B1^* ^‐^ *^ND1^* ^)^ + 2^(^ *^SERPINA1^* ^‐^ *^ND5^* ^)^)/2.

### 2.17 MitoTracker Green FM staining

Mitochondrial mass was evaluated using MitoTracker Green FM, which is a mitochondrial membrane potential–independent fluorescent dye. Cells were stained with MitoTracker Green FM (100 nM, 30 min, 37°C) and then subjected to NovoCyte flow cytometry at Ex/Em = 488/530 nm, with NovoExpress software (ACEA Biosciences) used for analysis.

Cells were then seeded onto coverslips for further visualization. After treatment, cells were stained with MitoTracker Green FM (200 nM, 30 min, 37°C) for mitochondrial staining and then incubated with Hoechst 33342 (1 μg/ml, 10 min, 25°C) for nuclear staining. Photomicrographs were captured using a DMi8 fluorescence microscope and Leica X software (Leica, 400 ×).

### 2.18 Reactive oxygen species assay

Cells were incubated in serum‐free medium with 2′,7′‐dichlorofluorescin diacetate (H2DCF‐DA, 10 μM) or MitoSOX (4 μM) for 30 min at 37°C and then collected to determine cellular and mitochondrial reactive oxygen species (ROS) levels. The fluorescence intensities of H2DCF‐DA and MitoSOX were measured via flow cytometry at Ex/Em = 488/530 and 488/572 nm, and the results were analysed using NovoExpress software (NovoCyte, ACEA Biosciences).

### 2.19 Mitochondrial membrane potential assay

The fluorescent dye tetramethylrhodamine ethyl ester (TMRE) was used to evaluate changes in mitochondrial membrane potential (MMP). After treatment, cells were collected and stained with TMRE (100 nM, 30 min, 37°C). The TMRE fluorescence was monitored via flow cytometry at Ex/Em = 488/572 nm with NovoExpress software (NovoCyte, ACEA Biosciences).

### 2.20 in vivo experiments

Female NOD/SCID mice (weight 18–22 g, six weeks old; Western Biotechnology Chongqing, China) were provided with water and standard laboratory food ad libitum and housed in a pathogen‐free environment with 12‐h light/dark cycle and 55% ± 10% humidity at a temperature of 20–25°C. The protocols were approved by the Ethical and Welfare Committee of Guizhou Medical University (approval no. 1800217) and performed under the National Institutes of Health Guide for the Care and Use of Laboratory Animals and in accordance with the declaration of Helsinki.

After one week of acclimatization, mice were anesthetized via CO_2_ inhalation, and a slow‐release oestrogen pellet (0.72 mg, 90‐day release) was implanted in the back neck region. The next day, mice were inoculated with 5.0 × 10^6^ MCF‐7TR cells (100 μl, PBS/Matrigel; v/v = 1:1) in the bilateral inguinal mammary fat pads. When the tumour volume reached 30–50 mm^3^, the mice were randomized into groups (*n* = 5) and were treated with or without TAM (20 mg/kg, i.g.) in the presence or absence of baicalein (30 mg/kg, i.p.), or olive oil solvent at an equal volume every three days for 30 days. Tumour size and body weight were then recorded every three days. The tumour volume was calculated as 1/2 × length × (width)^2^. After treatment, the mice were sacrificed after being anesthetized via CO_2_ inhalation. The tumour and visceral tissues were dissected for further evaluation.

### 2.21 Histological analysis

Immunohistochemistry (IHC) analysis was used to evaluate the expression of Ki‐67, HIF‐1α, PGC‐1α, LHDA and cleaved caspase‐3 in tumour tissue sections (5 μm) obtained after animal experiments. IHC analysis was carried out using an immunohistochemical stain detection kit. Protein expression was calculated as the mean OD (MOD) per unit area of tumour tissue (*n* = 5) using ImageJ software (v.1.8.0). The heart, lung, liver, spleen and kidney sections were stained with haematoxylin and eosin (H&E) and observed using a DMi8 microscope and Leica X software (Leica, 400 ×).

### 2.22 Detection of haematological parameters

Whole blood samples (0.2–0.3 ml per mouse) were collected by retro‐orbital sinus bleeding and added to tubes coated with EDTA dipotassium salt at 4°C, and the cells were assessed using an auto haematology analyser (MC‐6500; Icubio, Shenzhen, China).

### 2.23 Analysis of the association between HIF‐1α expression and TAM sensitivity

Z‐scores for the correlation of TAM sensitivity and HIF‐1α mRNA expression in the NCI‐60 cell line were obtained from CellMiner (http://discover.nci.nih.gov/cellminer/, v.2020.4, database: 2.5). The association between HIF‐1α expression and TAM sensitivity was assessed using regression analysis with the Spearman correlation.

### 2.24 Analysis using the Kaplan–Meier plotter database

Relapse‐free survival curves for patients with ER‐positive breast cancer were generated using an online open‐access analysis tool (http://kmplot.com/analysis/).^31^ Patients receiving endocrine therapy with TAM alone were selected. The patients were categorized as having high or low *HIF‐1α* gene expression (gene symbol: 200989_at) using the autoselect best cutoff mode. The *p* values were analysed using a log‐rank test.

### 2.25 Statistical analysis

All results were expressed as the mean ± standard deviation (SD) for at least three independent experiments. Differences between two groups were analysed using a two‐tailed Student's *t* test and differences among multiple groups were analysed using one‐way ANOVA followed by the Bonferroni post hoc test with GraphPad Prism 8.0 software (GraphPad Inc., San Diego, CA, USA). *p* < 0.05 was indicated statistically significant.

## 3 RESULTS

### 3.1 Baicalein sensitizes TAM‐resistant cells to OHT‐induced growth inhibition and apoptosis

**FIGURE 1:**
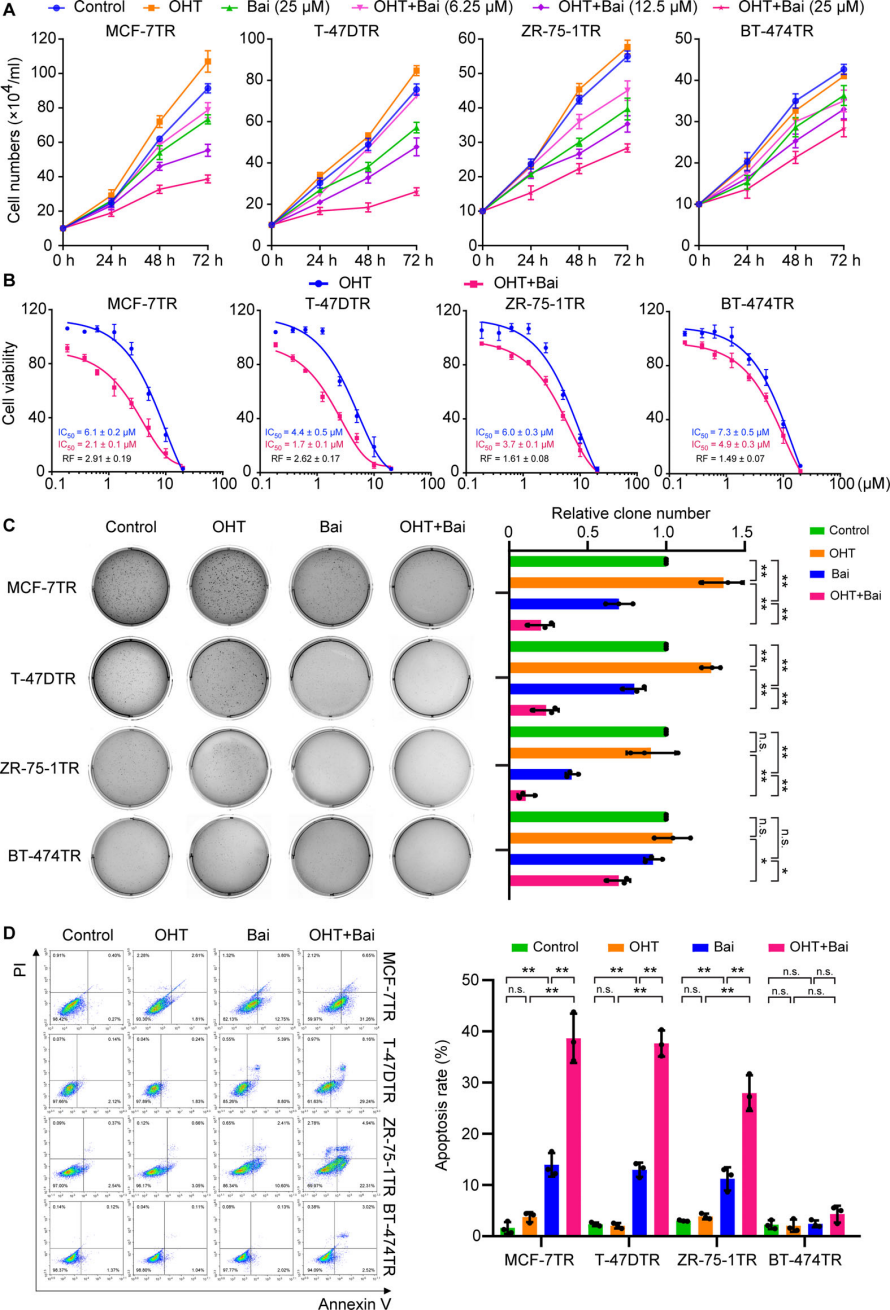
Baicalein enhances OHT‐induced growth inhibition and apoptosis in TAM‐resistant cells. (A) Cells were treated with OHT (1 μM) in the presence or absence of baicalein at the indicated concentrations for 24, 48 or 72 h, and growth curves were generated using the trypan blue dye exclusion assay (*n* = 3). (B) Cell viability was assessed using an MTT assay after cells were exposed to various concentrations of OHT in the presence or absence of baicalein (25 μM) for 48 h (*n* = 3). (C) Clonogenic cell survival was evaluated using a soft‐agar colony‐formation assay. The clones were cultured with or without OHT (1 μM) in the presence or absence of baicalein (25 μM) for 21 days and photographed using a ChemiDoc XRS^+^ system under a white light pattern (× 1). The number of clones was calculated using ImageJ software (*n* = 3). (D) Cell apoptosis was detected via flow cytometry. Apoptosis‐positive cells were defined as both early apoptotic (annexin V–positive and PI‐negative) and late apoptotic (annexin V–positive and PI‐positive) cells. Cells were treated with or without OHT (1 μM) in the presence or absence of baicalein (25 μM) for 48 h (*n* = 3). Data are presented as mean ± SD. *p < 0.05, **p < 0.01, control vs. baicalein (Bai); control vs. OHT; OHT vs. OHT plus baicalein; baicalein vs. OHT plus baicalein

We used four different TAM‐resistant cell lines (MCF‐7TR, T‐47DTR, ZR‐75‐1TR and BT‐474TR) to evaluate the efficacy of baicalein to enhance the sensitivity of resistant cells to TAM. We first monitored the growth of resistant cell lines treated with OHT (1 μM) in the presence or absence of various concentrations of baicalein. In these cells, OHT caused no inhibitory effects on cell growth, but the growth of cells treated with OHT and baicalein at 12.5 and 25 μM was significantly lower than that of cells treated with OHT alone (Figure 1A). We next measured cell viability using the MTT assay, plotted dose–response curves, and calculated the IC_50_ of OHT in the presence and absence of baicalein (25 μM). We found that the IC_50_ value decreased when OHT was combined with baicalein (Figure 1B). We then calculated the reversal fold (RF), which is defined as RF = IC_50_ (OHT)/ IC_50_ (OHT + baicalein). The RF values for baicalein in OHT‐treated MCF‐7TR, T‐47DTR, ZR‐75‐1TR and BT‐474TR cells were 2.91 ± 0.19, 2.62 ± 0.17, 1.61 ± 0.08 and 1.49 ± 0.07, respectively. Clonogenic growth was used to further verify the efficacy of baicalein and revealed that baicalein was able to reduce clonal numbers of MCF‐7TR, T‐47DTR and ZR‐75‐1TR cells. In addition, OHT co‐treatment with baicalein further reduced the clone numbers in these cells (Figure 1C). We next evaluated the impact of baicalein on OHT‐induced apoptosis using annexin V/PI staining. Consistently, baicalein induced 13.9% ± 1.8%, 13.0% ± 1.1% and 11.2% ± 1.9% apoptosis in MCF‐7TR, T‐47DTR and ZR‐75‐1TR cells, which increased to 38.7% ± 3.9%, 37.6% ± 2.1% and 27.9% ± 2.8%, respectively, when OHT (1 μM) and baicalein (25 μM) were combined. Thus, baicalein augmented the apoptotic effects of OHT in MCF‐7TR, T‐47DTR and ZR‐75‐1TR TAM‐resistant cells (Figure 1D). Because the RF values in MCF‐7TR and T‐47DTR cells were higher, the two cell types were used in the subsequent investigation. Our results demonstrate that baicalein sensitized cells to TAM and induced both apoptosis and inhibition of proliferation in the TAM‐resistant cells.

### 3.2 Baicalein suppresses HIF‐1α expression and transcriptional function in TAM‐resistant cells

**FIGURE 2:**
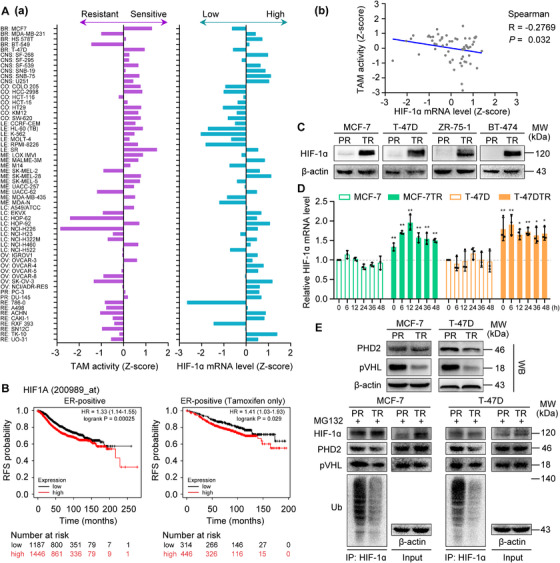
Relationship between HIF‐1α expression and TAM therapeutic response in cancer cells and breast cancer patients. (A) HIF‐1α expression is negatively correlated with TAM therapeutic response in NCI‐60 cells. (a) Left: Sensitivity of NCI‐60 cells to TAM is represented by the Z‐score, with cells categorized as sensitive (0 to 3) or resistant (0 to −3). Right: HIF‐1α expression in NCI‐60 cells is represented by the Z‐score, with expression categorized as high (0 to 3) or low (0 to −3). (b) HIF‐1α expression is inversely correlated with the sensitivity of NCI‐60 cancer cells to TAM (Spearman *R* = −0.2769; *p* = 0.032). (B) Relapse‐free survival curves for patients with ER‐positive breast cancer (*n* = 2633) and patients with ER‐positive breast cancer receiving TAM alone (*n* = 760) according to the Kaplan–Meier plotter database. *p* values and hazard ratios (HRs) are shown. (C) HIF‐1α expression determined in parental (PR) and TAM‐resistant (TR) cells, with β‐actin used as the loading control. (D) *HIF‐1α* mRNA levels in parental and TAM‐resistant cells. mRNA levels were measured at the time points indicated after the culture medium was refreshed. Results were normalized to *β‐actin* mRNA levels and presented as the fold change compared with parental cells at 0 h (n = 3). Data are shown as mean ± SD. *p < 0.05, **p < 0.01, parental cells vs. TAM‐resistant cells at the same time point. (E) Expression levels of PHD2 and pVHL, and ubiquitination of HIF‐1α in parental (PR) and TAM‐resistant (TR) cells. Cells were pretreated with 10 μM MG132 for 12 h.

It has been confirmed that increased HIF‐1α expression and activity are involved in TAM resistance in breast cancer cells.^11^, ^32^ These observations prompted us to determine a possible association between HIF‐1α expression and the TAM therapeutic response. We analysed this association in cancer cells and breast cancer patients, using the CellMiner tool and the Kaplan–Meier plotter database. We found that HIF‐1α expression is inversely correlated with the sensitivity of NCI‐60 cancer cells to TAM (Figure 2A). Kaplan–Meier plotter analysis demonstrated that high HIF‐1α expression was associated with shorter relapse‐free survival in a cohort of patients with ER‐positive breast cancer (*n* = 2633), and this subgroup of patients who received TAM as their only endocrine therapy (*n* = 760; Figure 2B). These results demonstrate that high HIF‐1α expression is strongly related to a poor TAM therapeutic response in cancer cells and patients with ER‐positive breast cancer. Consistently, we also found that the amount of HIF‐1α protein was clearly elevated in MCF‐7TR, T‐47DTR, ZR‐75‐1TR and BT‐474TR TAM‐resistant cells, even under normoxic conditions, but this was not observed in the corresponding parental cells (Figure 2C). Furthermore, *HIF‐1α* mRNA levels in MCF‐7TR and T‐47DTR cells were higher than in their parental cells (Figure 2D). HIF‐1α is hydroxylated by PHDs and then pVHL binds to hydroxylated‐HIF‐1α to mediate ubiquitination and facilitate its proteasomal degradation.^33^ The expression of PHD2 and pVHL, their interaction with HIF‐1α, and ubiquitination levels of HIF‐1α were significantly reduced in MCF‐7TR and T‐47DTR cells compared with their parental cells (Figure 2E). These results indicate that the increase in HIF‐1α in TAM‐resistant cells was due to enhanced *HIF‐1α* mRNA expression and reduced degradation of HIF‐1α protein.

**FIGURE 3:**
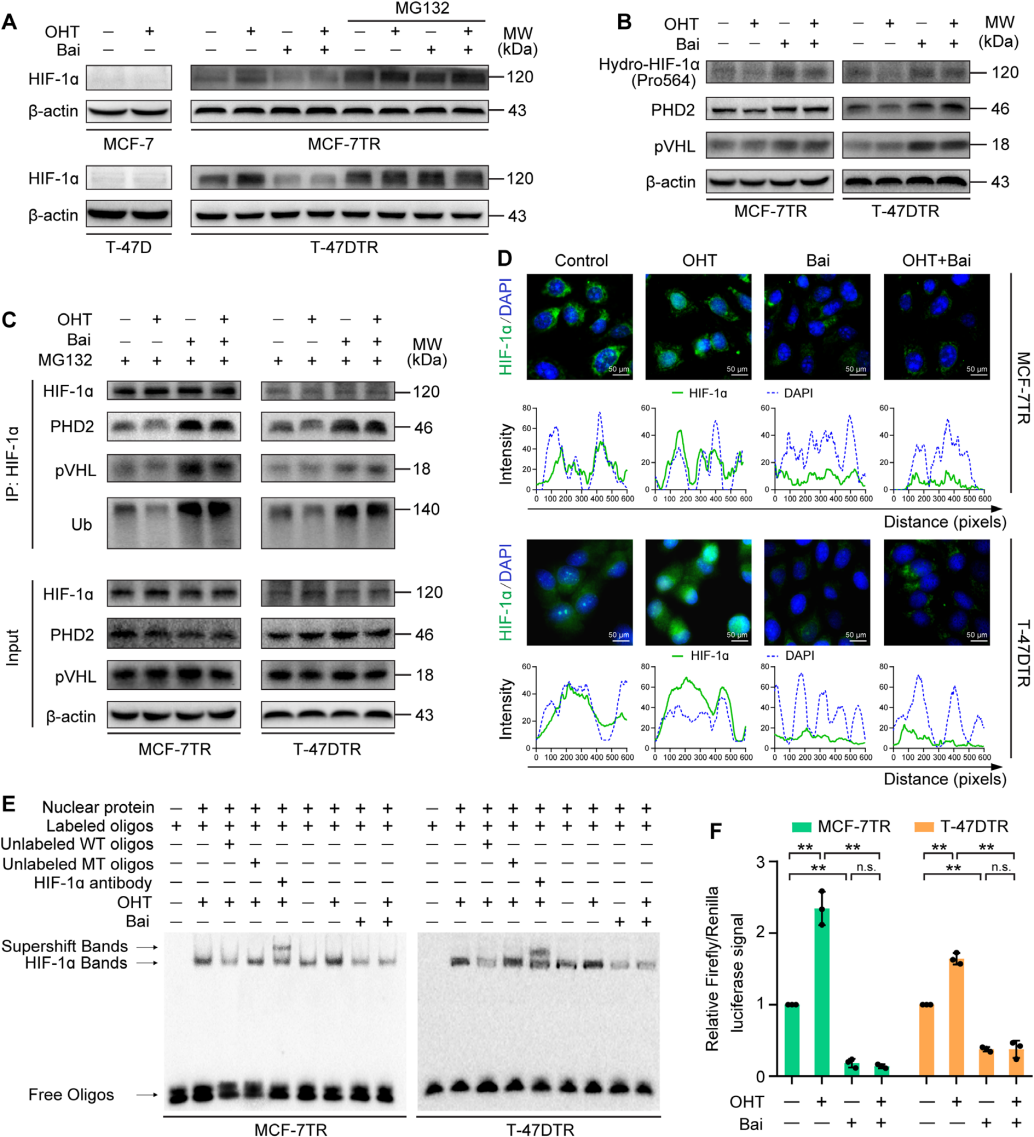
Baicalein reduces the expression and transcriptional activity of HIF‐1α in TAM‐resistant cells. Parental and TAM‐resistant cells were treated with OHT (1 μM), baicalein (25 μM), or OHT (1 μM) combined with baicalein (25 μM) for 48 h. TAM‐resistant cells were pretreated with 10 μM MG132 for 12 h and then treated as mentioned above. (A) HIF‐1α expression was detected via western blotting. (B) Expression levels of prolyl‐hydroxylated‐HIF‐1α (Pro564), PHD2 and pVHL were detected via western blotting. (C) Cell lysates were immunoprecipitated with HIF‐1α antibody. Immunoprecipitated materials and total cell extracts were analysed via western blotting with specific antibodies for ubiquitin (Ub), PHD2 and pVHL. Immunoprecipitated HIF‐1α was used as the loading control. (D) Subcellular localization of HIF‐1α was confirmed by immunofluorescence staining. HIF‐1α is represented by green fluorescence and cell nuclei were stained with DAPI (blue fluorescence; magnification, 400 × ; scale bars, 50 μm). Fluorescence intensity distribution curves show superposition of green fluorescence (HIF‐1α) and blue fluorescence (DAPI), confirming nuclear localization of HIF‐1α. (E) Baicalein inhibits the binding of HIF‐1α to a biotin‐labelled HRE consensus oligonucleotide in the presence or absence of OHT treatment. A specific anti‐HIF‐1α antibody was used to confirm HIF‐1α–HRE formation via a supershifted complex. Unlabelled wild‐type (WT) and mutant‐type (MT) probes were used as competitor oligonucleotides . (F) HRE‐luciferase reporter activity was measured using a dual‐luciferase assay. HRE‐luciferase reporter plasmids (pGL3‐HRE) and pRL Renilla luciferase control plasmids (ratio 10:1) were transfected into cells. After transfection, the cells were treated as mentioned above, and the dual‐luciferase assay was then performed using lysed cells (n = 3). Results are represented as mean ± SD. *p < 0.05, **p < 0.01, control vs. OHT; control vs. baicalein (Bai); OHT vs. OHT plus baicalein; baicalein vs. OHT plus baicalein

Furthermore, the expression and transcriptional activity of HIF‐1α were increased in the presence of OHT in MCF‐7TR and T‐47DTR cells (Figure 3A,E and F), which further indicates that the HIF‐1α signal was activated under OHT in TAM‐resistant cells. We next explored whether the inhibitory effects of OHT enhanced by baicalein may arise from HIF‐1α inhibition. HIF‐1α expression in MCF‐7TR and T‐47DTR cells was significantly downregulated in the presence of baicalein with or without OHT treatment (Figure 3A). We next determined how HIF‐1α expression was inhibited and found that *HIF‐1α* mRNA levels were unaffected by baicalein or OHT combined with baicalein (Figure S1A), and the baicalein or OHT combined with baicalein‐mediated reduction in HIF‐1α protein was not impacted by the protein synthesis inhibitor CHX, but was blocked by the proteasomal inhibitor MG132 (Figure S1B and Figure 3A), which indicates that HIF‐1α expression was inhibited via a post‐transcriptional regulatory mechanism. Pro402 and Pro564, two proline residues in the ODD domain of HIF‐1α, are the main target sites for hydroxylation by PHDs.^33^ We explored that baicalein or OHT combined with baicalein augmented the expression of prolyl‐hydroxylated‐HIF‐1α (Pro564), PHD2 and pVHL (Figure 3B). HIF‐1α was immunoprecipitated to demonstrate that its interaction with PHD2 or pVHL, and the extent of its ubiquitination, were enhanced by baicalein or OHT combined with baicalein (Figure 3C). Subsequently, we assessed the influence of baicalein or OHT combined with baicalein on the intracellular localization of HIF‐1α in MCF‐7TR and T‐47DTR cells using subcellular protein fractions and fluorescence staining. We observed that the nuclear translocation of HIF‐1α was reduced by baicalein or OHT combined with baicalein (Figure S1C and Figure 3D). Furthermore, the transcriptional activity of HIF‐1α was measured by the EMSA and HRE‐luciferase reporter assays. The results showed that the binding activity of HIF‐1α to the HRE and HRE‐Luc reporter activity were significantly suppressed by baicalein in both the presence and absence of OHT treatment (Figure 3E and F). Our results reveal that baicalein reduced HIF‐1α expression by promoting its degradation and inhibited its transcriptional activity, thus suppressing OHT‐induced HIF‐1α activation.

### 3.3 Baicalein reduces the elevated aerobic glycolysis in TAM‐resistant cells

**FIGURE 4:**
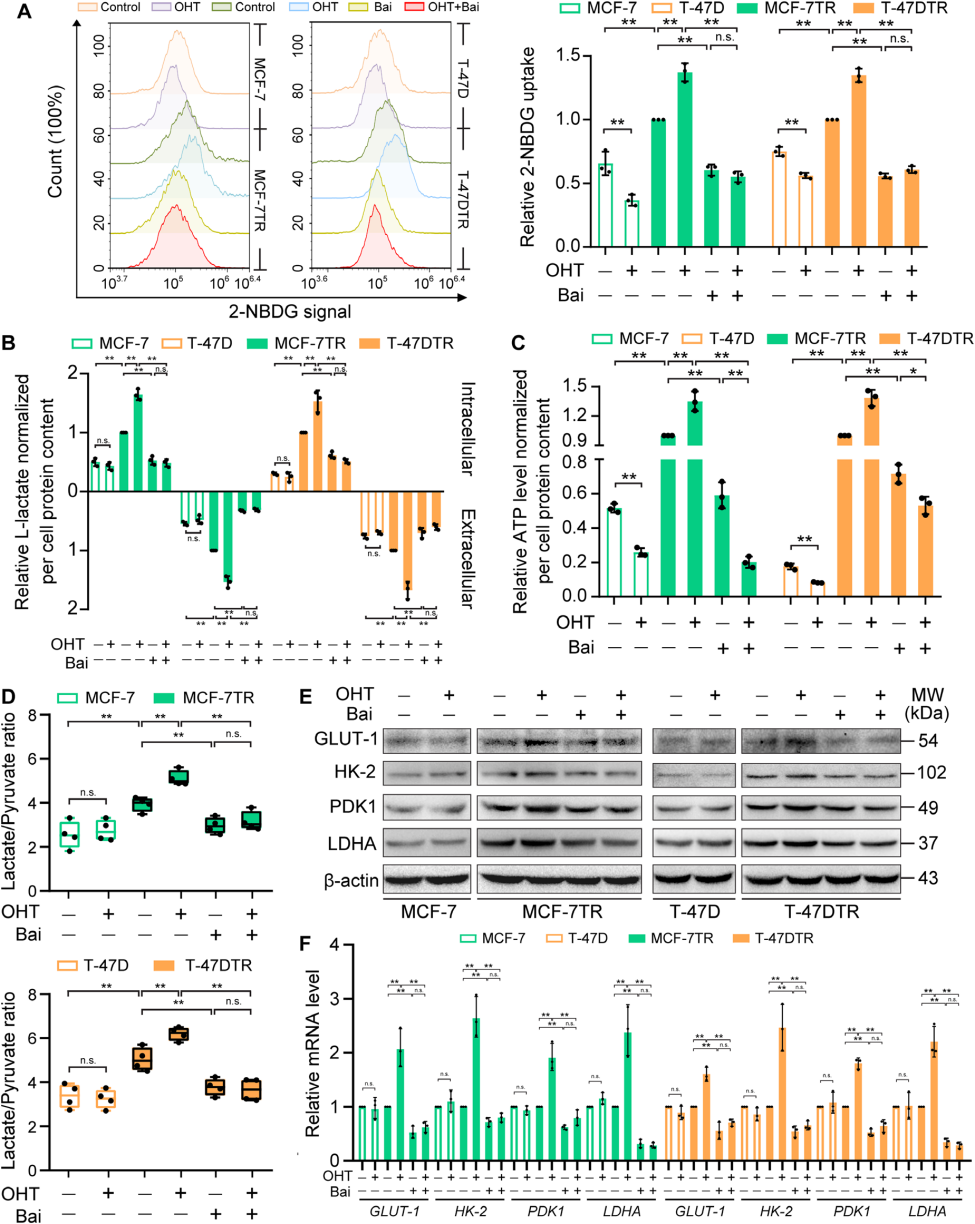
Baicalein inhibits aerobic glycolysis in TAM‐resistant cells. Parental and TAM‐resistant cells were treated with OHT (1 μM), baicalein (25 μM), or OHT (1 μM) combined with baicalein (25 μM) for 48 h. (A) Glucose uptake was measured via flow‐cytometric estimation of 2‐NBDG. Representative histogram overlays and relative 2‐NBDG signals show mean fluorescence intensities (*n* = 3). (B) Intracellular and extracellular levels of L‐lactate were measured using an L‐lactate assay kit. Lactate levels were normalized to the protein concentration (*n* = 3). (C) ATP content in cellular extracts was determined using an ATP detection kit. Intracellular ATP levels were normalized to the protein concentration (*n* = 3). (D) Lactate and pyruvate levels in parental and TAM‐resistant cells were measured using LC‐MS, and lactate/pyruvate ratios were calculated (*n* = 4). (E) Western blots of the expression of HIF‐1α–targeted glycolytic proteins, including GLUT‐1, HK‐2, PKD1 and LDHA. (F) mRNA levels of *GLUT‐1*, *HK‐2*, *PKD1* and *LDHA* detected via real‐time PCR. Results were normalized to *β‐actin* mRNA levels and reported as fold change in comparison with control cells (*n* = 3). Results are shown as mean ± SD. *p < 0.05, **p < 0.01, control (PR) vs. control (TR); control vs. OHT; control vs. baicalein (Bai); OHT vs. OHT plus baicalein; baicalein vs. OHT plus baicalein

TAM‐resistant cells exhibit enhanced aerobic glycolysis in comparison with parental cells,^17^ in a process that is mediated by HIF‐1α hyperactivation.^11^ We evaluated whether baicalein affects this aerobic glycolysis in TAM‐resistant cells. Our results revealed that OHT markedly decreased glucose consumption and ATP production in both MCF‐7 and T‐47D cells (Figure 4A and C), probably because of the OHT‐induced growth inhibition and an increase in apoptosis.^34^ Indeed, the rate of glucose uptake, intracellular and extracellular L‐lactate levels and the total amount of ATP generated were all clearly enhanced in MCF‐7TR and T‐47DTR cells in comparison with parental MCF‐7 and T‐47D cells, but baicalein inhibited aerobic glycolysis in the two TAM‐resistant cell lines (Figure 4A–C). Consistently, OHT enhanced glucose consumption, intracellular and extracellular L‐lactate levels and the total amount of ATP in MCF‐7TR and T‐47DTR cells, which were reduced by baicalein treatment (Figure 4A–C). In addition, measurement of lactate and pyruvate levels via LC‐MS showed that the lactate/pyruvate ratio was higher in MCF‐7TR and T‐47DTR cells in comparison with the parental cell lines, indicating that pyruvate was more effectively shunted towards aerobic glycolysis in the TAM‐resistant cells (Figure 4D). Furthermore, baicalein decreased the lactate/pyruvate ratio with or without OHT treatment in MCF‐7TR and T‐47DTR cells (Figure 4D). HIF‐1α transcriptionally regulates the expression of several glycolytic proteins.^35^ Among these glycolytic proteins, glucose transporter‐1 (GLUT‐1) increases glucose uptake and accelerates the rate of glycolysis; hexokinase‐2 (HK‐2) catalyses the phosphorylation of glucose, which is the first step in glycolysis; pyruvate dehydrogenase kinase 1 (PDK1) inactivates pyruvate dehydrogenase to restrict the transformation of pyruvate into acetyl‐CoA, and thus promotes pyruvate utilization in glycolysis; lactate dehydrogenase A (LDHA) ultimately converts pyruvate to lactate and ATP.^35^ It was observed that OHT exerted non‐specific effects on HIF‐1α–targeted glycolytic genes in parental MCF‐7 and T‐47D cells, but upregulated the expression of GLUT‐1, HK‐2, PDK1 and LDHA in MCF‐7TR and T‐47DTR cells (Figure 4E and F). In addition, baicalein inhibited the expression of these genes and reversed their OHT‐elevated expression in the two TAM‐resistant cell lines (Figure 4E and F). These results indicate that OHT significantly elevated aerobic glycolysis in TAM‐resistant cells, and that this effect was suppressed by baicalein treatment.

### 3.4 Baicalein reverses mitochondrial dysfunction in TAM‐resistant cells via increased mitochondrial biogenesis

**FIGURE 5:**
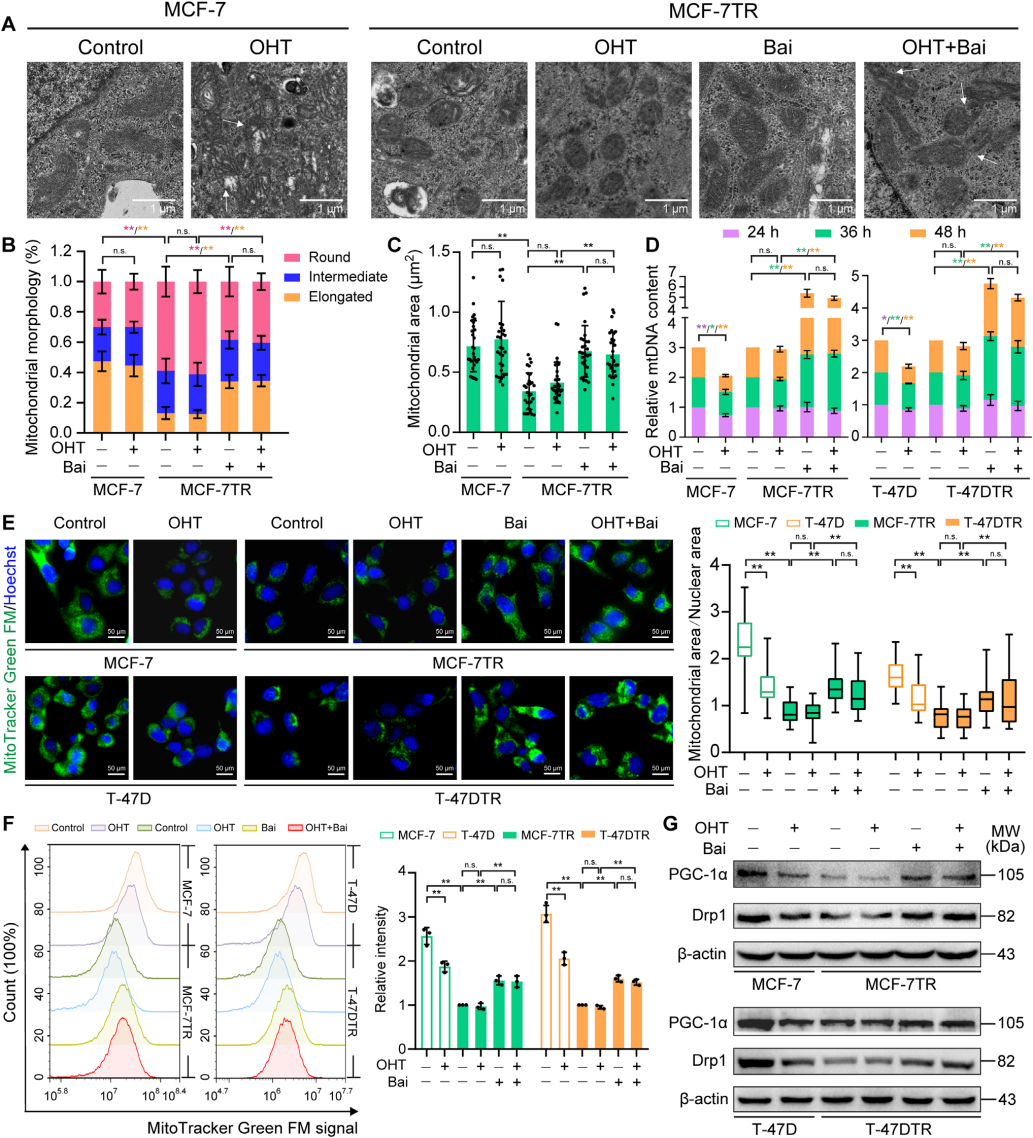
Baicalein increases mitochondrial biogenesis in TAM‐resistant cells. Parental and TAM‐resistant cells were treated with OHT (1 μM), baicalein (25 μM) or OHT (1 μM) combined with baicalein (25 μM) for 48 h. (A) Representative mitochondria in MCF‐7 and MCF‐7TR cells were observed via TEM (× 15 000 magnification; scale bar = 1 μm). White arrowheads indicate mitochondrial damage in MCF‐7 or MCF‐7TR cells and aggregation of mitochondria in MCF‐7TR cells. (B) The percentage of mitochondria with different morphologies was quantified (*n* = 6 cells). (C) Mitochondrial area was quantified using ImageJ software (*n* = 30 mitochondria). (D) Cells were treated for 24, 36 or 48 h, and mtDNA levels were assessed according to the relative levels of *ND1* and *ND5* in comparison with *SLCO2B1* and *SERPINA1* (*n* = 3). (E) Mitochondria were stained with MitoTracker Green FM (green fluorescence), and cell nuclei were stained with Hoechst 33342 (blue fluorescence; original magnification 400 × ; scale bars, 50 μm). The ratio of mitochondrial area to nuclear area was calculated (*n* = 30). (F) MitoTracker Green FM fluorescence was analysed via flow cytometry. Representative histogram overlays and relative MitoTracker Green FM fluorescence intensities. Mean fluorescence intensity values were calculated (*n* = 3). (G) Western blot analyses of PGC‐1α and Drp1 expression. Results are shown as mean ± SD. *p < 0.05, **p < 0.01, control (PR) vs. control (TR); control vs. OHT; control vs. baicalein (Bai); OHT vs. OHT plus baicalein; baicalein vs. OHT plus baicalein

Mitochondrial dysfunction contributes to the resistance to anticancer drugs seen in a variety of cancers, and recovery of mitochondrial function restores the susceptibility of resistant cells.^36^ Previous investigations have demonstrated that the mitochondria in breast cancer cells resistant to endocrine therapy exhibited a dysfunction, with a reduction in mtDNA levels and a dormant phenotype.^37^ Using TEM, we found that the number of mitochondria with an elongated shape and distinct cristae was higher in MCF‐7 cells, which reflects normal functional mitochondria (Figure 5A and B). However, most of the mitochondria in MCF‐7TR cells appeared round in shape, had fewer and shorter cristae and had a smaller area in comparison with MCF‐7 cells (Figure 5A–C). Thus, we investigated whether baicalein has an effect on the number and function of mitochondria. OHT treatment caused mitochondrial damage in parental MCF‐7 cells, but did not influence the shape of the mitochondria in MCF‐7TR cells (Figure 5A–C). However, baicalein treatment restored the mitochondrial morphology in MCF‐7TR cells to an appearance more like the one in parental MCF‐7 cells. Furthermore, OHT combined with baicalein also increased the number of elongated mitochondria and decreased the number of rounded mitochondria (Figure 5A–C). In addition, combined treatment with baicalein and OHT induced mitochondrial aggregation, indicating that the mitochondrial morphology changes occurred in the early stages of apoptosis (Figure 5A). Therefore, we investigated whether baicalein affects the number of mitochondria. In parental MCF‐7 and T47D cells, OHT reduced mtDNA levels, as shown by mitochondrial *ND1* and *ND5* gene copy numbers (Figure 5D), which is in line with previous research.^16^ By contrast, OHT had no obvious impact on mtDNA levels in MCF‐7TR and T‐47DTR cells, while baicalein conspicuously led to an increase in mtDNA levels from 36 to 48 h (Figure 5D). Baicalein also increased the number of mitochondria as determined using MitoTracker Green FM, a measure of mitochondrial mass (Figure 5E and F). In addition, mtDNA levels and mitochondrial numbers were also restored when given as co‐treatment with OHT and baicalein in MCF‐7TR and T‐47DTR cells (Figure 5D–F).

Peroxisome proliferator–activated receptor‐gamma coactivator 1α (PGC‐1α) is a positive regulator of mitochondrial biogenesis and function.^38^ In addition, dynamin‐related‐protein 1 (Drp1) is the major factor involved in stimulating mitochondrial fission, correcting the number of mitochondria during mitosis, and removing damaged mitochondrial fragments.^39^ Thus, we examined whether baicalein increased mtDNA levels and mitochondrial numbers through these two critical mediators of mitochondrial biogenesis and fission. We found that OHT decreased the expression of PGC‐1α and Drp1 in MCF‐7 and T‐47D cells but had no effect on the two proteins in the corresponding resistant cell lines (Figure 5G). However, baicalein upregulated PGC‐1α and Drp1 expression in MCF‐7TR and T‐47DTR cells in the presence or absence of OHT (Figure 5G). For mitochondrial fission, Drp1 is recruited from the cytosol and assembled in the mitochondria in a process that is mediated by mitochondrial outer membrane proteins, including mitochondrial fission factor (Mff), fission 1 (Fis1), and mitochondrial dynamics proteins of 49 kDa (MiD49) and 51 kDa (MiD51).^40^ It was observed that mitochondrial and total levels of Drp1 and Fis1, rather than Mff, MiD49 and MiD51, significantly increased after baicalein and combined baicalein and OHT treatment (Figure S2). These results show that mitochondrial fission and biogenesis are promoted in TAM‐resistant cells both under baicalein or OHT combined with baicalein treatment.

### 3.5 Baicalein increases OHT‐induced mitochondria‐mediated ROS, leading to apoptosis in TAM‐resistant cells

**FIGURE 6:**
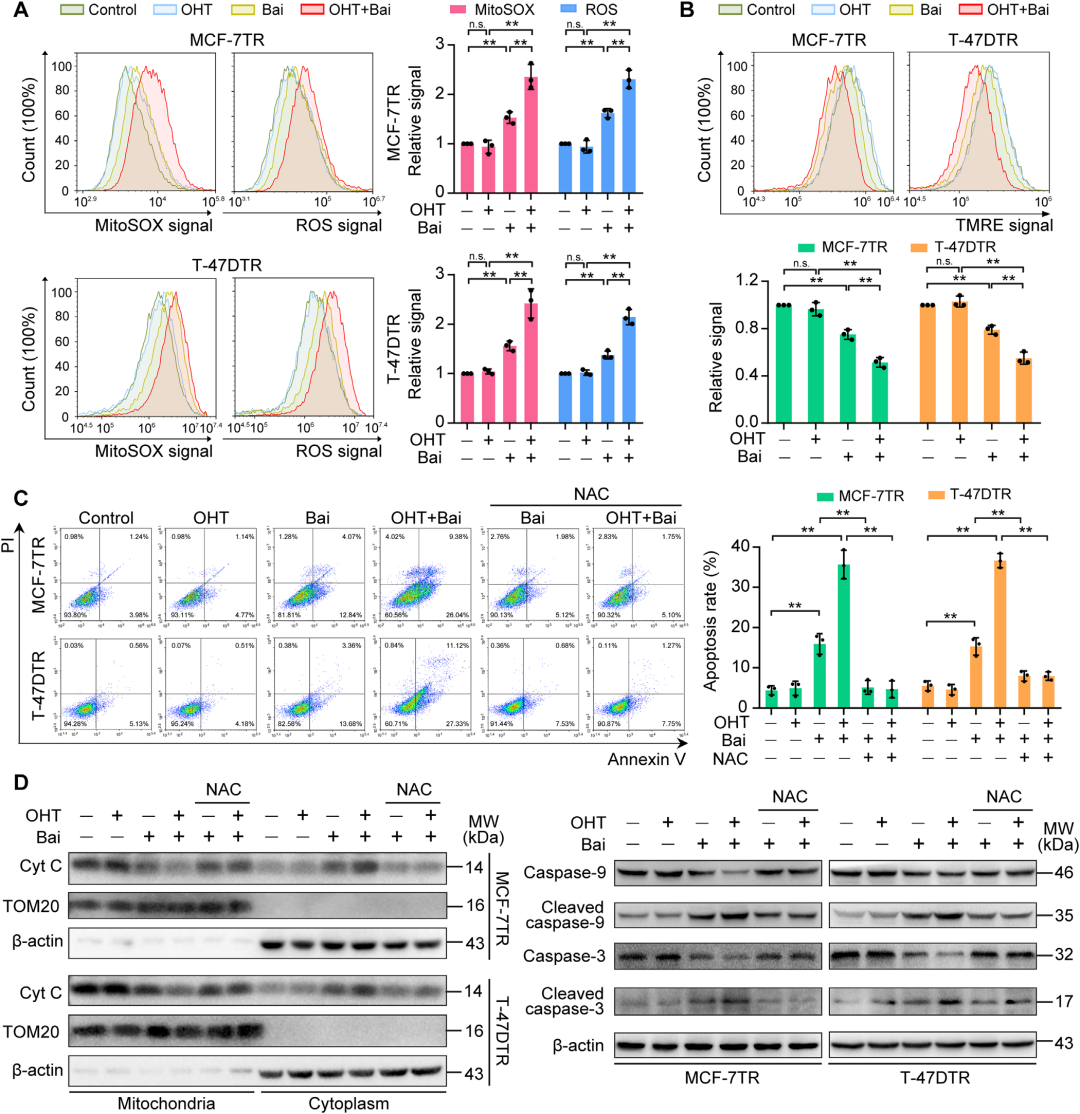
Baicalein increases OHT‐induced mitochondria‐mediated apoptosis in TAM‐resistant cells. Cells were treated with or without OHT (1 μM) in the presence of baicalein (25 μM) for 48 h. (A) Mitochondrial and intracellular ROS levels were evaluated using MitoSOX and H2DCF‐DA labelling, respectively, with detection via flow cytometry (*n* = 3). (B) The MMP was measured using TMRE staining and flow cytometry (*n* = 3). Cells were pretreated with the ROS scavenger NAC (10 mM) for 1 h and then treated with OHT (1 μM) in the presence of baicalein (25 μM) for a further 48 h. (C) Cell apoptosis was detected using annexin V and PI staining coupled with flow cytometry (*n* = 3). (D) Levels of cytochrome c (Cyt C) in mitochondria or cytoplasm, full‐length caspase‐9 and ‐3 and cleaved caspase‐9 and ‐3 were detected using western blotting. TOM20 and β‐actin were used as the loading control for mitochondrial fractions and cytosolic lysates, respectively. Results are presented as mean ± SD. *p < 0.05, **p < 0.01, control vs. baicalein (Bai); control vs. OHT; OHT vs. OHT plus baicalein; baicalein vs. OHT plus baicalein; baicalein vs. baicalein plus NAC; OHT plus baicalein vs. OHT plus baicalein plus NAC

Our findings suggesting that baicalein increases mitochondrial content prompted us to investigate whether the promotive effect of baicalein on OHT‐induced apoptosis in resistant cells was related to mitochondria‐mediated apoptotic events. We evaluated mitochondria‐mediated apoptotic events, including ROS accumulation, MMP reduction and expression profiles of key apoptotic proteins. Mitochondrial and intracellular ROS levels were measured using flow cytometry to determine MitoSOX‐Red and H2DCF‐DA fluorescence intensities. The results revealed that OHT had no significant influence on mitochondrial ROS in MCF‐7TR and T‐47DTR cells, but baicalein significantly increased mitochondrial ROS. This upregulation was further enhanced by OHT and baicalein co‐treatment in TAM‐resistant MCF‐7TR and T‐47DTR cells (Figure 6A). A similar result was obtained when we assessed changes in intracellular ROS, which were also increased by baicalein treatment and further enhanced by baicalein and OHT co‐treatment (Figure 6A). Furthermore, the results showed that baicalein treatment caused a slight reduction in MMP in both MCF‐7TR and T‐47DTR cells in comparison with the controls, as determined by TMRE staining (Figure 6B). The MMP level was further reduced after OHT and baicalein co‐treatment (Figure 6B). To further determine whether ROS generation contributed to the increase in OHT‐induced apoptosis, MCF‐7TR and T‐47DTR cells were pretreated with the ROS scavenger NAC. The results showed that NAC ameliorated the apoptosis induced by co‐treatment with baicalein and OHT (Figure 6C), indicating that the ROS generation was responsible for the increase in sensitivity of resistant cells. Furthermore, western blot results revealed that baicalein increased the expression of cytoplasmic cytochrome c and decreased mitochondrial cytochrome c (Figure 6D), and decreased the full‐length caspase‐9 and ‐3 levels, along with increases in the levels of cleaved caspase‐9 and ‐3 in MCF‐7TR and T‐47DTR cells, and these effects were further enhanced in OHT combined with baicalein (Figure 6D). Similarly, NAC alleviated the baicalein‐ and OHT combined with baicalein‐induced changes in cytochrome c, caspase‐9 and caspase‐3 (Figure 6D). Taken together, these results indicate that baicalein increased the number of mitochondria, which led to restoration of ROS generation and augmented OHT‐induced mitochondrial apoptosis in TAM‐resistant cells.

### 3.6 Stabilization of HIF‐1α reverses the resensitizing effect of baicalein on TAM‐resistant cells

**FIGURE 7:**
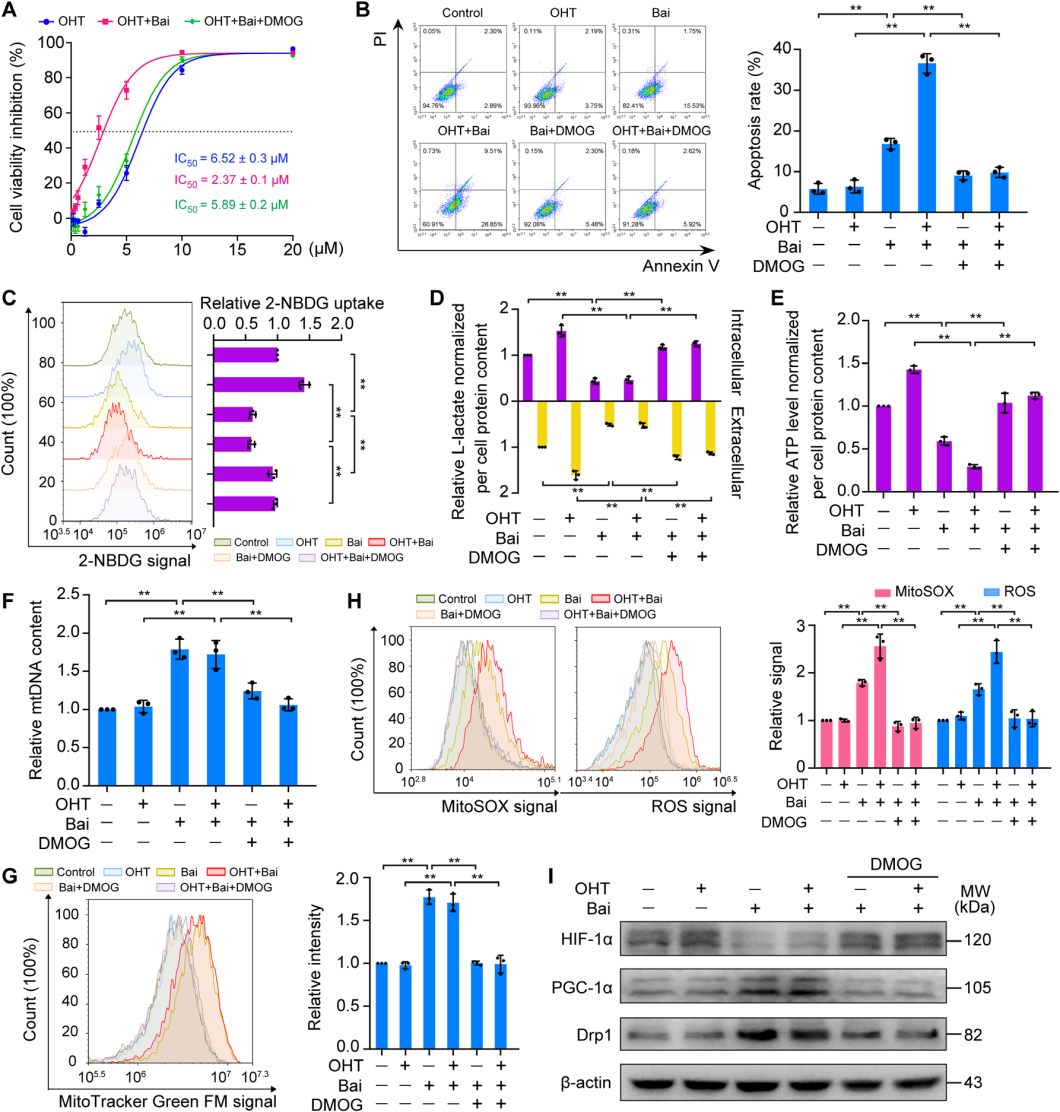
Stabilization of HIF‐1α reverses the resensitizing effect of baicalein on TAM‐resistant cells. MCF‐7TR cells were pretreated with DMOG (200 μM) for 4 h and then treated with or without OHT (1 μM) in the presence of baicalein (25 μM) for 48 h. (A) Reversible effects of DMOG on the combined OHT‐ and baicalein‐induced inhibition of MCF‐7TR cell viability. Cell viability was evaluated using an MTT assay (*n* = 3). (B) Reversible effects of DMOG on baicalein or combined OHT and baicalein‐induced apoptosis in MCF‐7TR cells. Cell apoptosis was measured using annexin V and PI staining followed by flow cytometry (*n* = 3). (C) Reversible effects of DMOG on baicalein‐ or combined OHT and baicalein‐induced inhibition of glucose uptake in MCF‐7TR cells. Glucose uptake was measured in terms of the 2‐NBDG signal on flow cytometry (*n* = 3). (D) Reversible effects of DMOG on the baicalein‐ or combined OHT and baicalein‐induced decrease in levels of intracellular and extracellular L‐lactate in MCF‐7TR cells (*n* = 3). Levels were determined using an L‐lactate assay kit. (E) Reversible effects of DMOG on baicalein‐ or combined OHT and baicalein‐induced reduction in ATP content in MCF‐7TR cells (*n* = 3). ATP content was measured using an ATP detection kit. (F) Reversible effects of DMOG on baicalein‐ or combined OHT and baicalein‐enhanced mtDNA content in MCF‐7TR cells. The mtDNA content was assessed by measuring *ND1* and *ND5* levels, with *SLCO2B1* and *SERPINA1* used as the reference genes (*n* = 3). (G) Reversible effects of DMOG on baicalein‐ or combined OHT and baicalein‐induced increase in mitochondrial number in MCF‐7TR cells. The number of mitochondria was evaluated using MitoTracker Green FM fluorescence labelling and flow cytometry (*n* = 3). (H) Reversible effects of DMOG on baicalein‐ or combined OHT and baicalein‐enhanced mitochondrial ROS production and intracellular ROS accumulation in MCF‐7TR cells. Mitochondrial and intracellular ROS levels were evaluated using MitoSOX and H2DCF‐DA labelling and detected via flow cytometry (*n* = 3). (I) Reversible effects of DMOG on baicalein‐ or combined OHT and baicalein‐enhanced expression of PGC‐1α and Drp1 in MCF‐7TR cells. Expression levels of PGC‐1α and Drp1 were analysed using western blotting. Results are presented as mean ± SD. *p < 0.05, **p < 0.01, control vs. baicalein (Bai); OHT vs. OHT plus baicalein; baicalein vs. baicalein plus DMOG; OHT plus baicalein vs. OHT plus baicalein plus DMOG

To determine whether HIF‐1α degradation is involved in the mechanism where baicalein promotes sensitization of cells to TAM, we performed rescue experiments in which the HIF‐1α stabilizer DMOG was applied to MCF‐7TR cells. DMOG inhibits PHD, which results in HIF‐1α stabilization and accumulation.^41^ Pretreatment with DMOG led to an effective increase in the levels of HIF‐1α and reversed the inhibitory effects of baicalein on HIF‐1α expression (Figure 7I). DMOG antagonized the sensitizing effects of baicalein on OHT‐induced inhibition of cell viability and elevation of cell apoptosis (Figure 7A and B).

We next attempted to confirm that baicalein‐mediated HIF‐1α degradation plays a role in reducing aerobic glycolysis and the recovery of mitochondrial function in MCF‐7TR cells. We found that DMOG offset the inhibitory effects of baicalein or OHT combined with baicalein on aerobic glycolysis, as reflected by increased glucose consumption, intracellular and extracellular lactate production, and ATP levels in MCF‐7TR cells (Figure 7C–E). Furthermore, baicalein or OHT combined with baicalein enhanced the amount of mtDNA and mitochondrial numbers compromised by pre‐incubation with DMOG in MCF‐7TR cells (Figure 7F and G). Consistent with the above results, mitochondrial and intracellular ROS production in MCF‐7TR cells treated with baicalein or OHT combined with baicalein also decreased in the presence of DMOG (Figure 7H). DMOG significantly decreased the enhanced PGC‐1α and Drp1 expression in MCF‐7TR cells treated with baicalein or OHT combined with baicalein (Figure 7I). Thus, our results indicate that baicalein inhibits aerobic glycolysis and restores the number and function of mitochondria, which increases the effects of OHT in TAM‐resistant cells via promotion of HIF‐1α degradation.

### 3.7 Baicalein increases the inhibitory effects of TAM on the growth of MCF‐7TR cells in vivo

**FIGURE 8:**
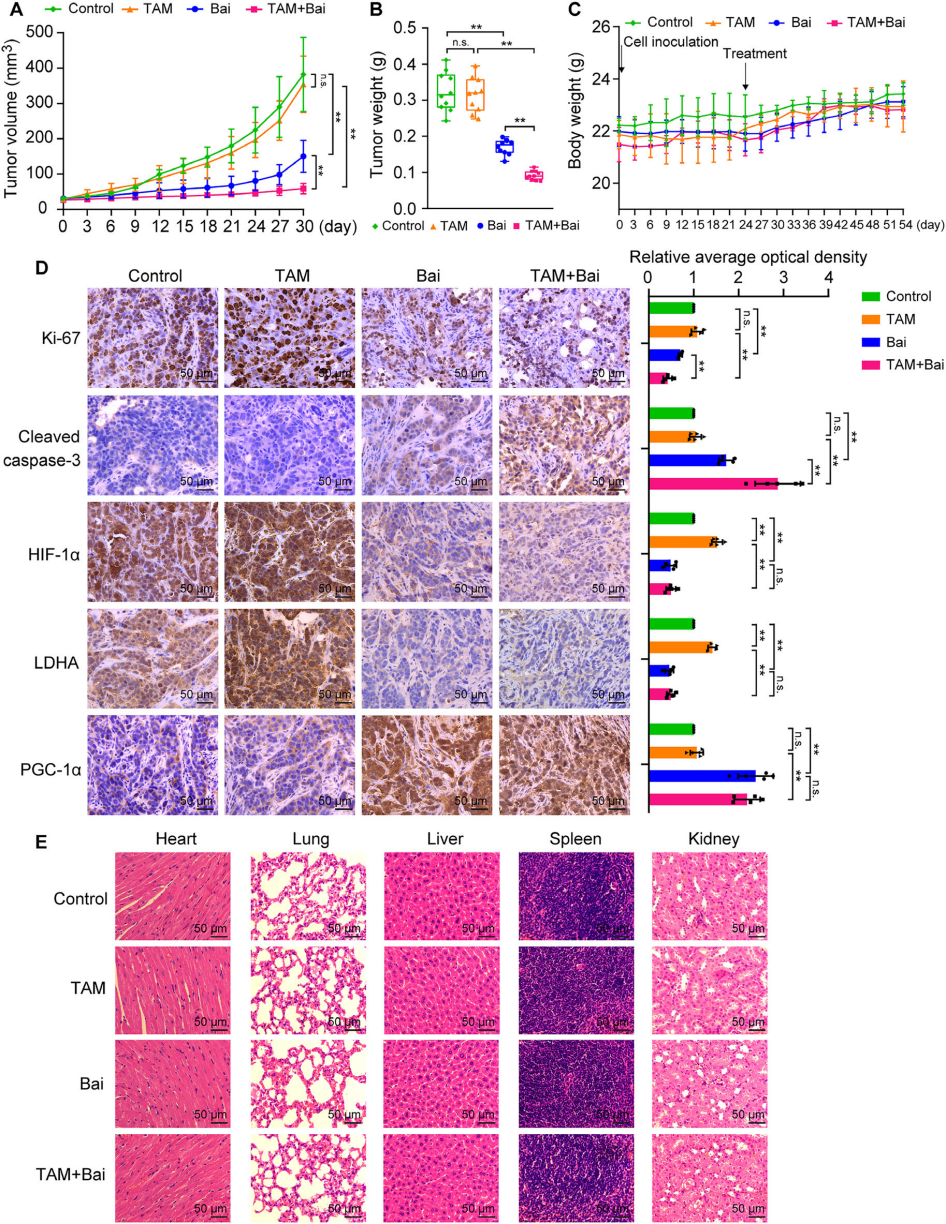
Baicalein increases the sensitivity of resistant cells to TAM in vivo. MCF‐7TR cells (5 × 10^6^ cells, PBS/Matrigel (v/v = 1:1), 100 μl) were inoculated into the bilateral inguinal mammary fat pads in NOD/SCID mice. When tumours grew to 30–50 mm^3^, the mice (*n* = 5 per group) were treated with or without TAM (20 mg/kg, i.g.) in the presence or absence of baicalein (30 mg/kg, i.p.), or an equal volume of olive oil solvent, every three days for 30 days. (A) Tumour growth curves for MCF‐7TR cells in NOD/SCID mice (*n* = 10). (B) Tumour weight for different treatment groups (*n* = 10). (C) Mean mouse body weight (*n* = 5). (D) Representative IHC images of Ki‐67, cleaved caspase‐3, HIF‐1α, LDHA, and PGC‐1α expression in tumour tissues (magnification, 400 ×). Scale bars, 50 μm. The average OD of the tumour areas was assessed using ImageJ software, and results are presented as the relative staining intensity (*n* = 5). (E) Representative H&E staining of heart, lung, liver, spleen and kidney sections (magnification, 400 ×). Scale bars, 50 μm. Results are presented as mean ± SD. *p < 0.05, **p < 0.01, control vs. TAM; control vs. baicalein (Bai); TAM vs. TAM plus baicalein; baicalein vs. TAM plus baicalein

We generated an MCF‐7TR–derived NOD/SCID mouse xenograft model to determine the ability of baicalein to increase TAM sensitivity in resistant cells in vivo. TAM was ineffective at inhibiting tumour growth relative to the control group (Figure 8A). In addition, TAM in combination with baicalein exerted a greater inhibitory effect than TAM alone, indicating reversal of resistance (Figure 8A). Furthermore, the average tumour weight was much lower in the TAM + baicalein group than in the TAM group (Figure 8B). The tumour inhibition rate by weight was calculated to be 2.8% in the TAM group, 47.3% in the baicalein group and 71.7% in the TAM + baicalein group.

We also evaluated cell proliferation and apoptosis in tumour tissues. We found that TAM combined with baicalein significantly inhibited tumour cell proliferation in comparison with TAM or baicalein alone, as shown by a reduction in Ki‐67 staining intensity (Figure 8D). TAM combined with baicalein also induced apoptosis in tumour cells, as indicated by an increase in cells positive for cleaved caspase‐3, as compared with TAM or baicalein alone (Figure 8D). Notably, baicalein or TAM combined with baicalein decreased the HIF‐1α expression in tumour tissues (Figure 8D). We confirmed the role of baicalein in increasing the sensitivity of resistant cells to TAM in vivo by demonstrating that the underlying mechanism involves inhibition of aerobic glycolysis and an increase in mitochondrial biosynthesis. We observed that LDHA expression levels were significantly downregulated whereas PGC‐1α expression levels were upregulated in tumour tissues from the baicalein and TAM + baicalein treatment groups (Figure 8D). Therefore, these in vivo studies demonstrated that TAM resistance can be reversed by baicalein, which results in restoration of the TAM‐induced apoptotic response and inhibition of cell proliferation in tumours.

### 3.8 Combined TAM and baicalein treatment has good biocompatibility

We evaluated the safety of all drug formulations by recording the body weight, haematological parameters and pathology of the major organs in the experimental mice. No significant loss of body weight (Figure 8C) or changes in haematological parameters (Table 1) were observed in any of the treatment groups. Furthermore, histological examination of the heart, lung, liver, spleen and kidneys revealed no observable differences among any of the treatment groups (Figure 8E). To further analyse the influence of baicalein or baicalein combined with TAM on cardiac, hepatic and renal functions, we administered the treatments to healthy female NOD/SCID and Kunming mice. There were no significant differences in functional cardiac parameters (left ventricular ejection fraction, systolic blood pressure, diastolic blood pressure, stroke volume and heart rate), liver function (alanine transaminase and aspartate aminotransferase) or kidney function (blood urea nitrogen, serum creatinine, and uric acid) between the control and treatment groups (Tables S2 and S3). These results indicate good biocompatibility of baicalein and TAM co‐treatment in vivo.

**TABLE 1:** Haematological parameters for female NOD/SCID mice following treatment with tamoxifen, baicalein or tamoxifen plus baicalein for 30 days. Data are presented as the mean ± SD (*n* = 5)

Haematological parameters	Control	Tamoxifen (20 mg/kg)	Baicalein (30 mg/kg)	Tamoxifen (20 mg/kg) + baicalein (30 mg/kg)	Standard
White blood cells (× 10^9^/L)	5.18 ± 0.83	4.76 ± 0.40	3.98 ± 1.02	3.28 ± 1.82	0.8–6.8
Lymphocytes (× 10^9^/L)	2.06 ± 0.46	2.32 ± 0.57	1.48 ± 0.89	1.14 ± 0.61	0.7–5.7
Monocytes (× 10^9^/L)	0.06 ± 0.05	0.02 ± 0.04	0.04 ± 0.05	0.02 ± 0.04	0.0–0.3
Granulocytes (× 10^9^/L)	0.82 ± 0.33	0.38 ± 0.15	0.30 ± 0.12	0.76 ± 0.32	0.1–1.8
Lymphocytes (%)	61.24 ± 5.46	62.90 ± 4.80	61.66 ± 2.56	62.24 ± 5.15	55.8–90.6
Monocytes (%)	4.08 ± 1.21	3.50 ± 1.49	3.46 ± 1.16	3.90 ± 1.01	1.8–6.0
Granulocytes (%)	33.16 ± 5.27	35.62 ± 2.71	30.06 ± 4.81	30.90 ± 4.00	8.6–38.9
Red blood cells (× 10^12^/L)	8.40 ± 0.49	7.85 ± 0.30	7.39 ± 0.58	7.71 ± 0.71	6.36–9.42
Haemoglobin (g/L)	132.6 ± 9.6	130.6 ± 10.6	134.0 ± 8.9	124.8 ± 13.4	110–143
Haematocrit (%)	41.14 ± 3.00	40.10 ± 3.09	41.80 ± 1.69	43.06 ± 1.23	34.6–44.6
Mean corpuscular volume (fl)	52.88 ± 0.75	52.42 ± 2.17	53.16 ± 1.09	52.70 ± 0.75	46.2–58.3
Mean corpuscular haemoglobin (pg)	17.48 ± 0.53	17.76 ± 0.93	16.96 ± 0.90	17.60 ± 0.80	15.8–19.0
Mean corpuscular haemoglobin concentration (g/L)	328.0 ± 11.6	335.6 ± 11.7	329.0 ± 11.7	342.4 ± 6.7	302–353
Red blood cell distribution width (%)	16.32 ± 0.27	15.70 ± 0.44	15.50 ± 0.57	16.16 ± 0.64	13.0–17.0
Platelet (× 10^9^/L)	960.4 ± 111.9	935.2 ± 60.2	750.4 ± 142.8	645.2 ± 128.6	450–1590
Mean platelet volume (fl)	5.16 ± 0.72	5.20 ± 0.75	4.88 ± 0.29	4.98 ± 0.38	3.8–6.0
Platelet distribution width (fl)	16.06 ± 0.18	17.06 ± 0.32	16.68 ± 0.49	16.22 ± 0.51	0.0–99.9
Plateletcrit (%)	0.53 ± 0.08	0.45 ± 0.01	0.47 ± 0.03	0.42 ± 0.17	0.0‐99.9

## 4 DISCUSSION

The emergence of resistance after initial therapy for cancer patients is almost inevitable and ultimately leads to treatment failure, which remains a significant clinical challenge.^42^ Although it is clear that resistant cancer cells often result in more aggressive tumour behaviour, metastasis, recurrence and poor clinical outcomes,^43^ it remains unclear how to best treat these resistant cells. Resensitization of therapy‐surviving cells to conventional drugs has been proposed as a promising strategy.^44^ Here we explored the potential of baicalein to resensitize resistant breast cancer cells to TAM both in vitro and in vivo. We used four TAM‐resistant cell lines and confirmed that baicalein improved the OHT suppression of cell proliferation and colony formation, promoted OHT‐induced apoptosis and enhanced TAM‐reduced tumour growth in the mammary fat pads of NOD/SCID mice. These results indicate that the use of baicalein as a sensitizer when in combination with TAM may represent a novel treatment for TAM‐resistant breast cancer cells.

High HIF‐1α mRNA levels were correlated with low TAM sensitivity in NCI‐60 cells by CellMiner analysis, and with poorer survival in patients with ER‐positive breast cancer who received TAM therapy by the Kaplan–Meier plotter analysis. Our study also revealed stable expression of HIF‐1α in four different TAM‐resistant cell lines, even under normoxic conditions, which is in agreement with previous investigations.^11^ The increase in HIF‐1α expression in TAM‐resistant cells can be attributed to both enhancement of mRNA levels and reduction in ubiquitin‐mediated degradation. In addition, aberrant expression and activation of HIF‐1α play a critical role in the onset of TAM resistance.^8^, ^11^ Identifying an inhibitor of HIF‐1α activation would be useful in the search for promising new agents to combat TAM resistance. Here, we found that baicalein clearly suppressed baseline and OHT‐induced HIF‐1α expression by promoting interaction between PHD2 and HIF‐1α, thereby increasing the prolyl hydroxylation of HIF‐1α, facilitating its binding to pVHL and stimulating ubiquitin‐mediated proteasomal degradation of HIF‐1α. Thus, baicalein inhibited nuclear translocation, binding to HRE and transcriptional activity of HIF‐1α. Our findings confirm that baicalein exerts potent inhibitory effects on HIF‐1α expression and activation in TAM‐resistant cells.

It is well documented that high levels of aerobic glycolysis enable cancer cells to lose sensitivity to treatment and contribute to the development of resistance to therapeutic agents.^45^ The enhanced glycolytic behaviour of resistant cancer cells leads to increased production and secretion of lactate, which renders the extracellular environment acidic, causing induction of multidrug resistance‐associated protein 1 expression, drug efflux and the generation of an apoptosis‐resistant phenotype.^46^ In addition, lactate activates its receptor GPR81, which induces PD‐L1 expression in cancer cells and thus provides an effective means of immune evasion.^47^ The high expression of key glycolytic enzymes in breast tumour tissues, such as HK‐2 and enolase‐alpha, represents a marker for poor clinical outcome during TAM therapy.^48^, ^49^ Suppression of aerobic glycolysis by metabolic inhibitors such as 2‐deoxyglucose and dichloroacetate, or by specific pharmacological or genetic inhibition of key glycolytic molecules, reduces cell proliferation and causes TAM sensitization in resistant breast cancer cells.^48^, ^49^, ^50^ It is now accepted that HIF‐1α is the primary driving force of the metabolic reprogramming seen in cancer cells, and constitutive activation of HIF‐1α resets the metabolic phenotype of the cell towards aerobic glycolysis by stimulating the glycolytic gene‐expression program.^51^ Our data show that TAM‐resistant cells exhibited excessive aerobic glycolysis in comparison with the parental cell lines, which was accompanied by HIF‐1α hyperactivation. However, baicalein effectively suppressed the aerobic glycolysis in TAM‐resistant cells, as indicated by reductions in glucose uptake, lactate production, ATP levels, and lactate/pyruvate ratio, along with downregulation of HIF‐1α–mediated expression of glycolytic genes, including *GLUT‐1*, *HK‐2*, *PDK1* and *LHDA*. Although OHT enhanced glycolysis in TAM‐resistant cells, OHT combined with baicalein reduced glycolysis to a level similar to that observed with baicalein treatment alone. Our rescue experiments showed that inhibition of aerobic glycolysis by baicalein or OHT combined with baicalein was prevented by DMOG. Thus, baicalein was able to suppress aerobic glycolysis via its ability to inhibit HIF‐1α expression and activation.

Mitochondria function as key regulators of many cellular processes and can sense the cellular environment, and ultimately determine whether a cell remains healthy or is diverted towards programmed cell death; therefore, mitochondria present a number of targets that may be utilized to affect drug‐induced cytotoxicity.^52^ Functional mitochondrial deficiency enables the cell to escape normal apoptotic pathways, which greatly contributes to drug resistance.^53^ Previous observations demonstrated that mitochondria in breast cancer cells resistant to endocrine therapy exhibit a dormancy phenotype associated with a reduction in mtDNA copy number, a decrease in mitochondrial biosynthesis and an impairment of OXPHOS.^37^ In agreement with these observations, our data revealed that mitochondria from TAM‐resistant cells showed a similar dormancy phenotype with morphological changes from a classically tubular shape and a branched network to a rounded fragmented shape, with reductions in mtDNA and mitochondrial numbers. Owing to the inhibitory effects of TAM on mitochondria,^13^, ^14^, ^15^, ^16^ TAM‐resistant cells contained fewer functional mitochondria in comparison with their parental cells. Thus, it seems that the cytotoxic potential of mitochondria was lost after long‐term exposure to OHT, and they could no longer affect OHT‐induced apoptosis.

Advances in our understanding of HIF‐1α biology have revealed that activation of HIF‐1α also leads to suppression of mitochondrial biogenesis and function. HIF‐1α negatively regulates mitochondrial biogenesis by mediating both repression of transcriptional activity and proteasome‐induced degradation of c‐Myc.^20^ HIF‐1α also transcriptionally activates the transcriptional repressor Hes‐related family BHLH transcription factor with YRPW motif 1 (HEY1), which in turn represses expression of *PTEN‐induced putative kinase 1* (*PINK1*), a gene essential for mitochondrial biogenesis, and therefore reduces mitochondrial mass and ROS accumulation.^54^ In addition, HIF‐1α mediates neuronal PAS domain protein 2 (NPAS2)‐suppressed mitochondrial biogenesis, which leads to metabolic reprogramming of OXPHOS to aerobic glycolysis via downregulation of PGC‐1α.^55^ Depletion of HIF‐1α using small interfering RNA restores cell clustering–reduced mitochondrial mass and ROS production.^56^

Prompted by the mitochondrial dysfunction seen in TAM‐resistant cells and the inhibitory effects of baicalein on HIF‐1α, we explored the possibility that baicalein could reverse TAM resistance by affecting mitochondria. In contrast to the fixed copy numbers of the nuclear genome, copies of mtDNA appear to fluctuate and are more plastic.^57^ Our results showed that baicalein significantly increased the mtDNA content in TAM‐resistant cells and that treatment with baicalein reversed the dominant morphological changes, increased the mitochondrial number and enhanced the expression of PGC‐1α and Drp1. Furthermore, mitochondria are important sources of ROS generation, and accumulation of high ROS levels induces cell apoptosis.^58^ As baicalein treatment upregulated the mitochondrial number, OHT in combination with baicalein resulted in significant mitochondria‐associated ROS generation and exacerbated intracellular ROS accumulation, causing loss of MMP and leading to apoptosis in TAM‐resistant cells. Importantly, the active oxygen scavenger NAC reversed the pro‐apoptotic effect of combined baicalein and TAM treatment of TAM‐resistant cells, implying that ROS are the critical mediator involved in this apoptotic effect. These findings demonstrate that baicalein increased the sensitivity of resistant cells to OHT by increasing their mitochondrial content and restoring mitochondrial function, thereby generating ROS, which enhanced OHT‐induced apoptosis. It therefore appears that restoring the number of functional mitochondria to induce surplus ROS is an alternative approach for increasing the TAM sensitivity of resistant cancer cells. Furthermore, baicalein and baicalein combined with OHT increased the levels of Drp1 and Fis1 in mitochondria, indicating enhancement of mitochondrial fission. It has been proposed that mitochondrial fission participates in stimulating apoptosis,^59^ and previous evidence suggested that Drp1 and Fis1 are able to facilitate cytochrome c release during mitochondrial fission, leading to activation of the mitochondrial apoptosis pathway.^60^ Therefore, further investigation is needed to explore the detailed mechanism of action for baicalein combined with OHT in mitochondrial fission and apoptosis. Our rescue experiments showed that the baicalein‐mediated increases in mitochondrial biogenesis and ROS generation were blocked by DMOG, and that OHT combined with baicalein‐induced mitochondrial biogenesis, ROS generation and apoptosis were also reversed. These results provide convincing evidence that inhibition of HIF‐1α by baicalein can restore mitochondrial function during resensitization of resistant cells to TAM.

Our animal experiments not only proved a role for baicalein as an enhancer of the inhibitory efficacy of TAM for resistant cells in vivo but also demonstrated a lack of toxic effect when baicalein and TAM were combined. Co‐treatment with baicalein and TAM was well tolerated in NOD/SCID mice and did not cause a loss of body weight; in addition, there was no toxic effect on peripheral blood cells and primary organs. Cardiac, hepatic and renal functions in healthy NOD/SCID mice and Kunming mice were also unaffected by combined baicalein and TAM treatment. In addition, pharmacokinetic data show that the oral bioavailability of TAM is enhanced by co‐administration with baicalein, as indicated by an increase in the peak plasma TAM concentration and the area of plasma concentration–time curve in rats.^61^ This effect might be due to baicalein‐mediated inhibition of CYP3A in the liver and the P‐glycoprotein efflux pump in the small intestine, which reduce total body clearance of TAM.^61^ Importantly, the low toxicity and enhanced bioavailability of baicalein mean that its combined regimen with TAM can represent a novel strategy for treating TAM‐resistant breast cancer.

Our data clearly show that baicalein can reduce HIF‐1α expression and transcriptional activity, which results in inhibition of HIF‐1α–mediated glycolysis and restores mitochondrial numbers and their ability to generate ROS. These beneficial effects ultimately help to enhance both OHT‐mediated inhibition of proliferation and induction of apoptosis, thereby resensitizing resistant cells to TAM. Finally, our findings suggest a promising beneficial effect of combining TAM with baicalein for patients with TAM‐resistant cancer and highlight HIF‐1α as an attractive target for restoring TAM sensitivity.

## CONFLICT OF INTEREST

The authors declare that they have no conflict of interest.

## Supporting information

Supporting information

Supporting information

Supporting information

Supporting information

Supporting information

Supporting information
