# Supplementary figures and images for: Baicalein resensitizes tamoxifen‐resistant breast cancer cells by reducing aerobic glycolysis and reversing mitochondrial dysfunction via inhibition of hypoxia‐inducible factor‐1α
Source: Clin Transl Med. 2021 Nov 4;11(11):e577. doi: 10.1002/ctm2.577 (PMC8567056; doi:10.1002/ctm2.577)

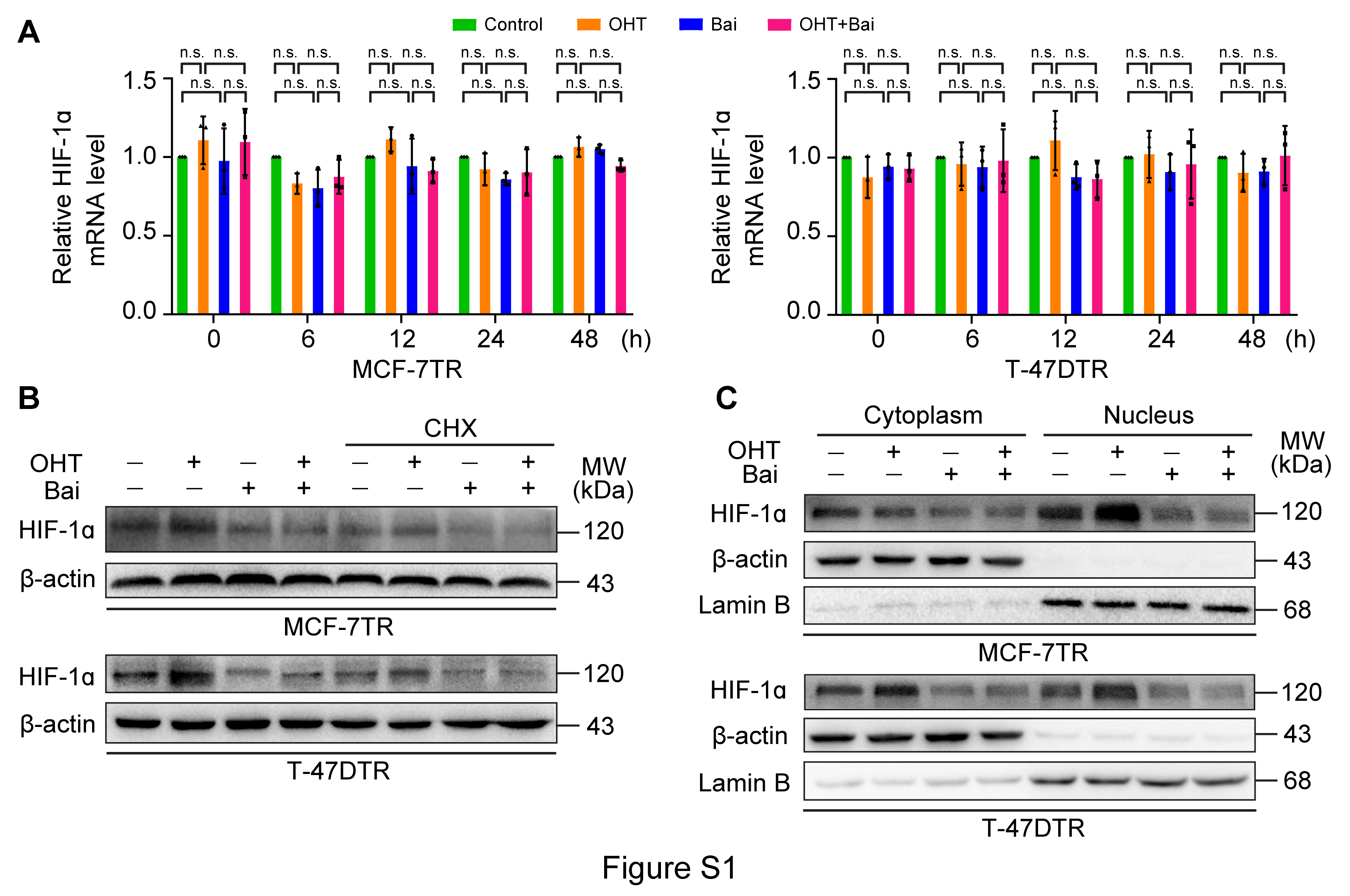

Supplement: Supplementary file 1 — Supporting information [file CTM2-11-e577-s001.tif]

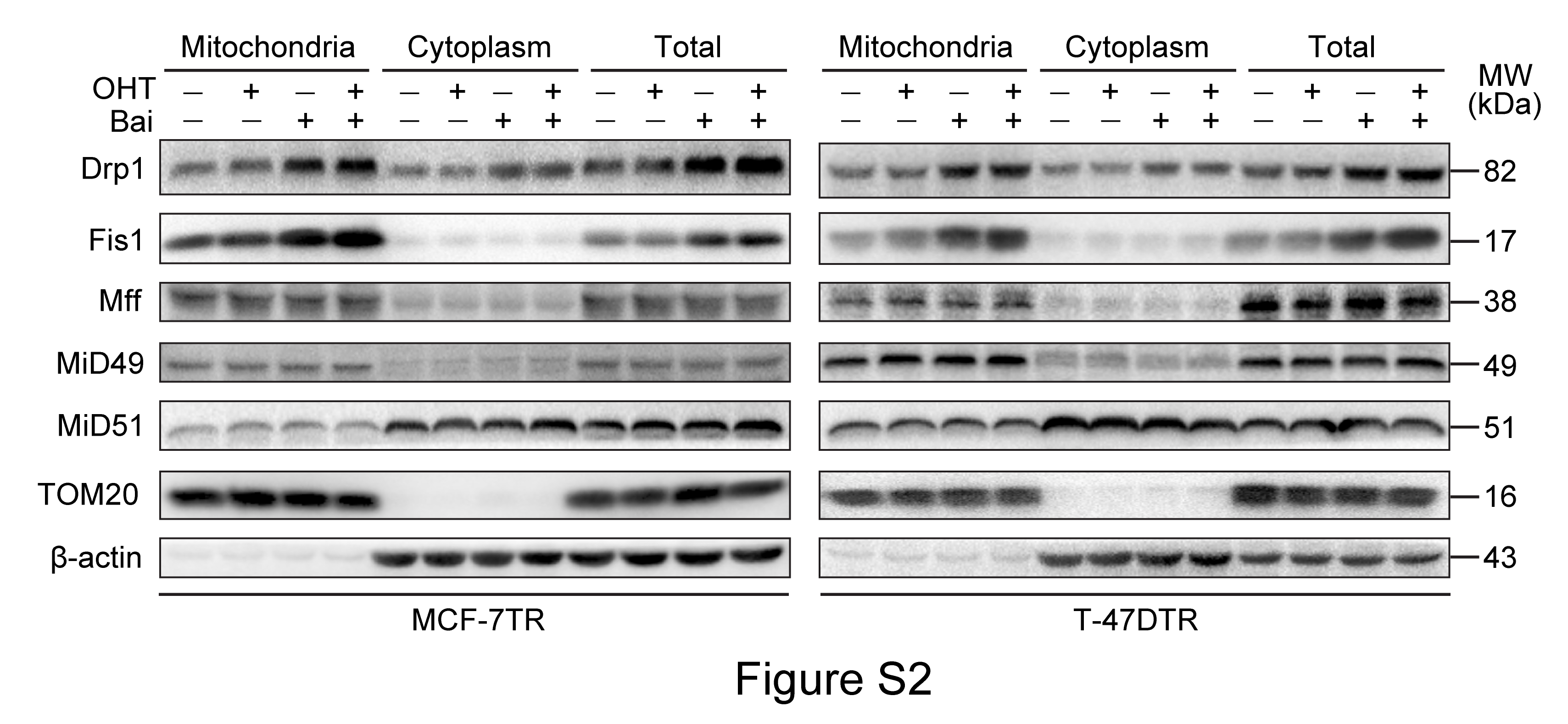

Supplement: Supplementary file 2 — Supporting information [file CTM2-11-e577-s004.tif]
