# Supplementary material for: Baicalein resensitizes tamoxifen‐resistant breast cancer cells by reducing aerobic glycolysis and reversing mitochondrial dysfunction via inhibition of hypoxia‐inducible factor‐1α
Source: Clin Transl Med. 2021 Nov 4;11(11):e577. doi: 10.1002/ctm2.577 (PMC8567056; doi:10.1002/ctm2.577)
Supplement: Supplementary file 3 — Supporting information [file CTM2-11-e577-s006.docx]

**Key material table**

| MATERIAL | SOURCE | IDENTIFIER |
| --- | --- | --- |
| Regents |  |  |
| Baicalein | Sigma–Aldrich | Cat#465119 |
| Tamoxifen | Sigma–Aldrich | Cat#T5648 |
| N-Acetyl-L-cysteine (NAC) | Sigma–Aldrich | Cat#A9165 |
| 4-hydroxytamoxifen (OHT) | Abcam | Cat#ab143638 |
| Dimethyloxallyl Glycine (DMOG) | Med Chem Express | Cat#HY-15893 |
| Z-Leu-Leu-Leu-al (MG132) | Sigma–Aldrich | Cat#M7449 |
| Cycloheximide (CHX) | Med Chem Express | Cat#HY-12320 |
| 3-(4,5-dimethylthiazol-2-yl)-2,5-diphenyltetrazolium bromide (MTT) | Solarbio | Cat#M8180 |
| Matrigel | Corning | Cat#356234 |
| SeaPlaque^®^ Agarose | Lonza | Cat#50101 |
| Radio Immunoprecipitation Assay Lysis Buffer | Beyotime | Cat#P0013K |
| Phenylmethanesulfonyl fluoride (PMSF) | Beyotime | Cat#ST505 |
| Immobilon Western Chemiluminescent HRP Substrate | MerckMillipore | Cat#WBKLS0500 |
| Protease Inhibitor Cocktail, EDTA-free (100×, PIC) | TransGen Biotech | Cat#DI101 |
| 2-(4-Amidinophenyl)-6-indolecarbamidine dihydrochloride (DAPI) | Bio-World | Cat#BD5010 |
| TB Green^®^ Premix Ex Taq™II (Tli RNaseH Plus) | TaKaRa | Cat#RR820A |
| Lipofectamine™ 2000 Transfection Reagent | Invitrogen | Cat#11668019 |
| Fluorescent glucose analog 2-Nitrobenzodeoxyglucose (2-NBDG) | Invitrogen | Cat#N13195 |
| MitoTracker™ Green FM | Thermo Fisher Scientific | Cat#M7514 |
| Hoechst 33342 Staining Solution | Beyotime | Cat# C1028 |
| 2',7'-Dichlorodihydrofluorescein diacetate  (H2DCFDA) | Med Chem Express | Cat#HY-D0940 |
| MitoSOX™ Red Mitochondrial Superoxide Indicator | Invitrogen | Cat#M36008 |
| Olive oil | Sigma | Cat #1478265 |
| Antibodies | SOURCE | IDENTIFIER |
| Monoclonal Rabbit anti-HIF-1α (D1S7W) | Cell Signaling Technology | Cat#36169 |
| Monoclonal Rabbit anti-PDK1 (EPR19573) | Abcam | Cat#ab207450 |
| Polyclonal Rabbit anti-PGC1 alpha | Abcam | Cat#ab54481 |
| Monoclonal Rabbit anti-Drp1 (D8H5) | Cell Signaling Technology | Cat#5391 |
| Monoclonal Rabbit anti-Hexokinase II (C64G5) | Cell Signaling Technology | Cat#2867 |
| Monoclonal Rabbit anti-Glut1 (D3J3A) | Cell Signaling Technology | Cat#12939 |
| Monoclonal Rabbit anti-LDHA (C4B5) | Cell Signaling Technology | Cat#3582 |
| Monoclonal Rabbit anti-Cleaved Caspase-3 (Asp175) (5A1E) | Cell Signaling Technology | Cat#9664 |
| Monoclonal Rabbit anti-Cleaved Caspase-9 (Asp315) (D8I9E) | Cell Signaling Technology | Cat#20750 |
| Monoclonal Mouse anti-Cytochrome c | Proteintech | Cat#66264-1-Ig |
| Monoclonal Mouse anti-Caspase-9 | Proteintech | Cat#66169-1-Ig |
| Monoclonal Mouse anti-Caspase-3 | Proteintech | Cat#66470-2-Ig |
| Monoclonal Mouse anti-β-Actin (4D3) | Bio-World | Cat#BS6007M |
| Polyclonal Rabbit anti-Lamin B (L75) | Bio-World | Cat#BS3547 |
| Polyclonal Rabbit anti-FIS1 | Proteintech | Cat#10956-1-AP |
| Polyclonal Rabbit anti-SMCR7/MID49 | Proteintech | Cat#28718-1-AP |
| Polyclonal Rabbit anti-SMCR7L/MID51 | Proteintech | Cat#20164-1-AP |
| Polyclonal Rabbit anti-MFF | Proteintech | Cat#17090-1-AP |
| Polyclonal Rabbit anti-TOM20 | Proteintech | Cat#11802-1-AP |
| Polyclonal Rabbit anti-pVHL | Proteintech | Cat#24756-1-AP |
| Monoclonal Rabbit anti-PHD2 [EPR3660(B)(2)] | Abcam | Cat#ab133630 |
| Monoclonal Mouse anti-Ubiquitin (P4D1) | Cell Signaling Technology | Cat#3936 |
| Monoclonal Mouse anti-Ki-67 (8D5) | Cell Signaling Technology | Cat#9449 |
| Monoclonal Rabbit anti-Hydroxy-HIF-1α (Pro564) (D43B5) | Cell Signaling Technology | Cat#3434 |
| Goat anti-Mouse IgG (H+L) HRP | Bio-World | Cat#BS12478 |
| Goat anti-Rabbit IgG (H+L) HRP | Bio-World | Cat#BS13278 |
| Goat polyclonal Secondary Antibody to Rabbit IgG - H&L (Alexa Fluor® 488) | Abcam | Cat#ab150077 |
| Kits | SOURCE | IDENTIFIER |
| Annexin V/PI staining kit | NanJing KeyGen Biotech | Cat#KGA107 |
| Nuclear and Cytoplasmic Protein Extraction Kit | Beyotime | Cat#P0028 |
| Cell Mitochondria Isolation Kit | Beyotime | Cat#C3601 |
| Pierce™ Bicinchoninic Acid (BCA) Protein Assay Kit | Thermo Fisher Scientific | Cat#23227 |
| Immunoprecipitation Kit (Protein A/G plus Agarose) | Sangon Biotech | Cat#C600689 |
| MiniBEST Universal RNA Extraction Kit | TaKaRa | Cat#9767 |
| PrimeScript^TM^ RT reagent Kit with gDNA Eraser (Perfect Real Time) | TaKaRa | Cat#RR047A |
| Chemiluminescent EMSA Kit | Beyotime | Cat#GS009 |
| SuperSignal^TM^ West Femto Maximum Sensitivity Substrate Kit | Thermo Fisher Scientific | Cat#34096 |
| Dual-Luciferase^®^ Reporter Assay System | Promega | Cat#E1910 |
| L-Lactate Assay Kit (Colorimetric/Fluorometric) | Abcam | Cat#ab65330 |
| ATP Determination Kit | Invitrogen | Cat#A22066 |
| MiniBEST Universal Genomic DNA Extraction Kit | TaKaRa | Cat#9765 |
| Human Mitochondrial DNA (mtDNA) Monitoring Primer Set | TaKaRa | Cat#7246 |
| Tetramethylrhodamine Ethyl Ester (TMRE)-Mitochondrial Membrane Potential Assay Kit | Abcam | Cat#ab113852 |
| Immunohistochemical stain detection kit | Zhongshan Golden Bridge Biotechnology | Cat#PV-9000 |
| Hematoxylin and Eosin (H&E) Staining Kit | Beyotime | Cat#C0105S |
| Plasmids | SOURCE | IDENTIFIER |
| pGL3-HRE luciferase reporter plasmid | Addgene | Cat #26731 |
| pRL-TK vector plasmid | Promega | Cat #E2241 |

**Cell culture**

The MCF-7TR and BT-474TR cell lines were cultured in Dulbecco’s minimum essential medium (DMEM; Gibco, Carlsbad, CA, USA) containing 10% fetal bovine serum (FBS; Gibco) and 10 μg/mL insulin (Sigma). T-47DTR cell line was cultured in Roswell Park Memorial Institute (RPMI)-1640 medium (Gibco) supplemented with 10% FBS and 10 μg/mL insulin. ZR-75-1TR cell line was cultured in RPMI-1640 medium supplemented with 10% FBS. The MCF-7, T-47D, and BT-474 cell lines were cultured in DMEM/F-12 (Gibco) supplemented with 10% FBS, whereas ZR-75-1 cells were cultured in RPMI-1640 medium supplemented with 10% FBS. All cells were incubated under 5% CO_2_ at 37°C.

**Measurement of lactate and pyruvate levels**

The intracellular metabolites were extracted using aliquots of water (200 μL/sample) and then freeze thawing three times. Then, 200 μL methanol containing ^13^C_6_-L-phenylalanine (200 ng/mL, as an internal standard) was added into the 50 μL cell lysate. The suspension was vortexed for 3 min, and then centrifuged twice at 15000 ×g for 10 min at 4 °C. The levels of lactate and pyruvate were detected using liquid chromatography-tandem mass spectrometry (LC-MS) methodology. The LC separation was achieved by using a SCIEX ExionLC system (AB SCIEX. Ltd., MA, USA) and MS detection was performed using a Triple Quad 5500 system (AB SCIEX). The separation was achieved on an ACQUITY UPLC^®^HSS T3 column (2.1×150 mm, 1.8 μm, Waters, MA, USA) at a flow rate of 0.2 mL/min. The LC mobile phase consisted of (A) water containing 5 mM ammonium acetate and 0.05% ammonia solution (25%) and (B) acetonitrile. The elution gradients were shown in Table 1 and the m/z and retention times for lactate and pyruvate are listed in Table 2.

Table 1 Gradient elution

| Time | Module | Events | Parameter |
| --- | --- | --- | --- |
| 0.00 | Pumps | Pump B Conc. | 10 |
| 2.00 | Pumps | Pump B Conc. | 20 |
| 6.00 | Pumps | Pump B Conc. | 90 |
| 7.00 | Pumps | Pump B Conc. | 90 |
| 7.10 | Pumps | Pump B Conc. | 10 |
| 12.00 | Pumps | Pump B Conc. | 10 |

Table 2 m/z and retention time of the lactate and pyruvate

| Compounds | m/z | Retention time |
| --- | --- | --- |
| Lactate | 89.000→43.000 | 1.48 |
| Pyruvate | 87.000→43.000 | 1.46 |

**Effect of treatment on the function of heart, liver, and kidney**

Healthy female NOD/SCID mice and female Kunming mice were purchased from Jrdun Biotechnology (Shanghai, China), raised in a pathogen-free or clean environment, and kept at a temperature of 20–25 °C and a humidity of 55% ± 10% with a 12-h light/dark cycle. These mice were included in each group (n = 3), and treated with or without TAM (20 mg/kg, i.g.) in the presence or absence of baicalein (30 mg/kg, i.p.), or an equal volume of olive oil solvent every 3 days for 30 days. The functional parameters of the heart, liver, and kidney in mice were observed every 10 days for up to 30 days after the mice were anesthetized using via CO_2_ inhalation. Blood samples (0.2-0.3 mL per mouse) were collected by retro-orbital sinus bleeding. All experiments were approved by the Ethical and Welfare Committee of Guizhou Medical university (approval no. 2100129), and the protocols were performed under the National Institutes of Health guide for the care and use of Laboratory animals and were in accordance with the Declaration of Helsinki.

Functional parameters of the heart, including left ventricular ejection fraction (LVEF), heart rate (HR), stroke volume, systolic blood pressure, and diastolic blood pressure were detected using Vevo2100 echocardiography (Visual Sonics, Toronto, Canada). The functional parameters of the liver, including alanine aminotransferase (ALT) and aspartate aminotransferase (AST) were analyzed using ALT (C009-2-1) and AST assay kits (C010-2-1). The functional parameters of the kidney, including urea nitrogen (BUN), serum creatinine (Cr), and uric acid (UA), were analyzed using urea (C013-1-1), creatinine (C011-2-1) and UA assay kits (C012-2-1). All kits were purchased from Nanjing Jiancheng Bioengineering Institute (Jiangsu, China), and these parameters were detected using a spectraMax M5/M5e microplate spectrophotometer (Molecular Devices, San Jose, CA, USA).

**Supplementary figures**

***Figure S1 Messenger RNA and protein levels for HIF-1α in MCF-7TR and T-47DTR cells.***

(A) The mRNA levels were detected after exposure to OHT (1 μM), baicalein (25 μM), or OHT (1 μM) combined with baicalein (25 μM) for 48 h. Results were normalized to *β-actin* mRNA levels and are represented as fold changes compared to control cells (n = 3). (B) TAM-resistant cells were pretreated with 8 μM CHX for 3 h, and then treated with OHT (1 μM), baicalein (25 μM), or OHT (1 μM) combined with baicalein (25 μM) for 48 h. (C) HIF-1α localization was analyzed from the cytoplasmic and nuclear fractions, referenced with β-actin and lamin B, respectively. Data are shown as the mean ± SD for three independent experiments. **P* < 0.05, ***P* < 0.01, control *vs.* OHT; control *vs.* baicalein (Bai); OHT *vs.* OHT plus baicalein; baicalein *vs.* OHT plus baicalein.

***Figure S2 Expression of mitochondrial fusion proteins in MCF-7TR and T-47DTR cells***.

The levels of Drp1, Fis1, Mff, MiD49 and MiD51 from mitochondrial fractions or cytosolic or total lysates were detected by western blotting. TOM20 and β-actin were used as loading controls for the mitochondrial fractions and cytosolic/total lysates respectively.
