# Supplementary material for: Baicalein resensitizes tamoxifen‐resistant breast cancer cells by reducing aerobic glycolysis and reversing mitochondrial dysfunction via inhibition of hypoxia‐inducible factor‐1α
Source: Clin Transl Med. 2021 Nov 4;11(11):e577. doi: 10.1002/ctm2.577 (PMC8567056; doi:10.1002/ctm2.577)
Supplement: Supplementary file 4 — Supporting information [file CTM2-11-e577-s002.docx]

**Table S1 Primer sequences for real-time PCR**

| Name | 5’-3’ sequence | Reference |
| --- | --- | --- |
| *HIF-1α* forward | TCC GAT GGA AGC ACT AGA CAA AG | [1](#_ENREF_1) |
| *HIF-1α* reverse | TGA CAA CTG ATC GAA GGA ACG TAA |  |
| *GLUT-1* forward | GGG GTC CTA TAA ACG CTA CGG | [2](#_ENREF_2) |
| *GLUT-1* reverse | GGG GGC ATT GAT GAC TCC AG |  |
| *HK-2* forward | TGG AGC GAG GTC TGA GCA AG | [3](#_ENREF_3) |
| *HK-2* reverse | ACC AGC AGG ACC CGG AAA TT |  |
| *PDK1* forward | CTG TGA TAC GGA TCA GAA ACC G | [4](#_ENREF_4) |
| *PDK1* reverse | TCC ACC AAA CAA TAA AGA GTG CT |  |
| *LDHA* forward | AGG CTA CAC ATC CTG GGC TAT | [5](#_ENREF_5) |
| *LDHA* reverse | CCC AAA ATG CAA GGA ACA CTA |  |
| *β-actin* forward | AGT TGC GTT ACA CCC TTT C | [6](#_ENREF_6) |
| *β-actin* reverse | CCT TCA CCG TTC CAG TTT |  |

**References**

1. Zhou L, Wang Y, Zhou M, et al. HOXA9 inhibits HIF-1alpha-mediated glycolysis through interacting with CRIP2 to repress cutaneous squamous cell carcinoma development. *Nat Commun*. 2018;9(1):1480.

2. Sur S, Nakanishi H, Flaveny C, et al. Inhibition of the key metabolic pathways, glycolysis and lipogenesis, of oral cancer by bitter melon extract. *Cell Commun Signal*. 2019;17(1):131.

3. Fang E, Wang X, Wang J, et al. Therapeutic targeting of YY1/MZF1 axis by MZF1-uPEP inhibits aerobic glycolysis and neuroblastoma progression. *Theranostics*. 2020;10(4):1555-1571.

4. Chen J, Cao S, Situ B, et al. Metabolic reprogramming-based characterization of circulating tumor cells in prostate cancer. *J Exp Clin Cancer Res*. 2018;37(1):127.

5. Zhang H, Li L, Chen Q, et al. PGC1beta regulates multiple myeloma tumor growth through LDHA-mediated glycolytic metabolism. *Mol Oncol*. 2018;12(9):1579-1595.

6. Ferreira E, Cronje MJ. Selection of suitable reference genes for quantitative real-time PCR in apoptosis-induced MCF-7 breast cancer cells. *Mol Biotechnol*. 2012;50(2):121-128.
