# Supplementary material for: Baicalein resensitizes tamoxifen‐resistant breast cancer cells by reducing aerobic glycolysis and reversing mitochondrial dysfunction via inhibition of hypoxia‐inducible factor‐1α
Source: Clin Transl Med. 2021 Nov 4;11(11):e577. doi: 10.1002/ctm2.577 (PMC8567056; doi:10.1002/ctm2.577)
Supplement: Supplementary file 5 — Supporting information [file CTM2-11-e577-s003.docx]

Table S2 The functional parameters of heart, liver and kidney of female NOD/SCID mice following treatment with or without tamoxifen (20 mg/kg/3 d, TAM) in the presence or absence of baicalein (30 mg/kg/3 d, Bai) for 30 days. The data are shown as the mean ± SD (n = 3).

| Treatment Time | 0 d | | | | 10 d | | | | 20 d | | | | 30 d | | | |
| --- | --- | --- | --- | --- | --- | --- | --- | --- | --- | --- | --- | --- | --- | --- | --- | --- |
| Treatment Group | Control | TAM | Bai | TAM+Bai | Control | TAM | Bai | TAM+Bai | Control | TAM | Bai | TAM+Bai | Control | TAM | Bai | TAM+Bai |
| Heart Markers | | | | | | | | | | | | | | | | |
| Left Ventricular Ejection Fraction (LVEF) | 0.72  ±0.01 | 0.70  ±0.01 | 0.67  ±0.05 | 0.68  ±0.02 | 0.68  ±0.07 | 0.66  ±0.03 | 0.74  ±0.03 | 0.72  ±0.02 | 0.72  ±0.02 | 0.69  ±0.02 | 0.72  ±0.02 | 0.71  ±0.01 | 0.71  ±0.02 | 0.72  ±0.01 | 0.69  ±0.02 | 0.73  ±0.01 |
| Systolic Blood Pressure (SBP, mmHg) | 108.8  ±1.6 | 111.0  ±4.1 | 109.4  ±3.8 | 114.7  ±2.5 | 120.7  ±7.1 | 116.1  ±3.9 | 111.2  ±2.8 | 116.1  ±11.1 | 116.7  ±1.7 | 122.3  ±7.2 | 121.6  ±7.7 | 119.6  ±1.6 | 110.8  ±8.0 | 114.9  ±1.0 | 116.2  ±2.4 | 110.2  ±4.1 |
| Diastolic Blood Pressure  (DBP, mmHg) | 69.4  ±3.6 | 75.2  ±4.2 | 71.7  ±3.4 | 72.4  ±3.9 | 72.7  ±5.3 | 70.7  ±2.9 | 73.9  ±1.5 | 74.0  ±6.0 | 72.3  ±1.6 | 74.3  ±2.4 | 73.7  ±5.6 | 76.8  ±0.2 | 65.5  ±0.3 | 71.3  ±3.3 | 70.7  ±4.0 | 68.0  ±1.8 |
| Stroke Volume  (SV, μL) | 30.2  ±3.3 | 27.5  ±2.2 | 28.5  ±4.0 | 29.1  ±4.3 | 32.9  ±5.1 | 31.7  ±8.2 | 36.4  ±8.0 | 36.9  ±1.0 | 33.2  ±0.9 | 36.5  ±3.5 | 36.3  ±4.6 | 35.7  ±1.8 | 36.4  ±4.5 | 40.8  ±2.1 | 36.5  ±3.5 | 38.6  ±5.5 |
| Heart Rate  (Bpm) | 420.3  ±13.9 | 422.0  ±10.4 | 426.7  ±9.0 | 413.3  ±3.4 | 419.7  ±13.9 | 421.0  ±2.2 | 419.0  ±12.8 | 429.7  ±7.1 | 435.3  ±6.0 | 424.7  ±15.7 | 429.0  ±7.8 | 425.3  ±2.5 | 421.3  ±3.3 | 417.3  ±10.5 | 417.3  ±5.2 | 416.3  ±4.6 |
| Liver Markers (Blood sample) | | | | | | | | | | | | | | | | |
| Alanine Transaminase (ALT, IU/L) | 50.5  ±3.8 | 46.2  ±1.1 | 47.5  ±0.9 | 46.1  ±1.8 | 46.9  ±2.3 | 49.5  ±5.3 | 49.6  ±3.3 | 46.3  ±2.8 | 51.1  ±6.1 | 51.0  ±5.0 | 53.1  ±5.1 | 57.4  ±7.9 | 49.9  ±2.1 | 45.5  ±4.7 | 51.7  ±7.1 | 45.7  ±3.9 |
| Aspartate Aminotransferase (AST, IU/L) | 144.1  ±11.2 | 130.9  ±7.9 | 139.7  ±13.6 | 145.5  ±15.5 | 149.0  ±18.2 | 135.6  ±10.7 | 134.1  ±7.4 | 132.2  ±13.2 | 132.8  ±10.2 | 130.2  ±6.5 | 125.1  ±12.5 | 124.1  ±9.2 | 124.3  ±5.7 | 129.0  ±11.4 | 122.9  ±5.0 | 131.8  ±5.8 |
| Kidney Markers (Blood sample) | | | | | | | | | | | | | | | | |
| Blood Urea Nitrogen (BUN, mM/L) | 15.2  ±1.0 | 16.1  ±0.6 | 14.0  ±1.9 | 15.1  ±1.6 | 13.8  ±1.8 | 14.7  ±0.8 | 15.1  ±0.6 | 14.9  ±0.2 | 14.3  ±0.9 | 14.5  ±0.5 | 14.6  ±0.9 | 14.9  ±1.0 | 13.9  ±0.7 | 14.4  ±0.5 | 14.9  ±1.2 | 14.8  ±0.8 |
| Serum Creatinine (Scr, μM/L) | 108.9  ±3.9 | 110.4  ±3.7 | 117.9  ±10.5 | 114.3  ±17.7 | 133.6  ±3.3 | 127.4  ±4.0 | 129.8  ±13.9 | 133.3  ±19.4 | 132.1  ±19.3 | 131.0  ±11.6 | 122.3  ±12.1 | 128.9  ±17.3 | 135.7  ±19.7 | 119.3  ±15.7 | 118.8  ±21.3 | 129.5  ±18.1 |
| Uric Acid  (UA, mM/L) | 54.1  ±5.8 | 57.4  ±1.8 | 56.6  ±1.5 | 57.0  ±5.8 | 48.2  ±3.4 | 54.3  ±2.9 | 51.1  ±1.9 | 51.5  ±1.9 | 55.3  ±3.9 | 58.0  ±7.7 | 59.3  ±8.4 | 52.7  ±1.4 | 59.0  ±2.9 | 53.9  ±0.9 | 56.5  ±3.9 | 51.6  ±6.1 |
