# Supplementary material for: Baicalein resensitizes tamoxifen‐resistant breast cancer cells by reducing aerobic glycolysis and reversing mitochondrial dysfunction via inhibition of hypoxia‐inducible factor‐1α
Source: Clin Transl Med. 2021 Nov 4;11(11):e577. doi: 10.1002/ctm2.577 (PMC8567056; doi:10.1002/ctm2.577)
Supplement: Supplementary file 6 — Supporting information [file CTM2-11-e577-s005.docx]

Table S3 The functional parameters of heart, liver and kidney of female Kunming mice following treatment with or without tamoxifen (20 mg/kg/3 d, TAM) in the presence or absence of baicalein (30 mg/kg/3 d, Bai) for 30 days. The data are shown as the mean ± SD (n = 3).

| Treatment Time | 0 d | | | | 10 d | | | | 20 d | | | | 30 d | | | |
| --- | --- | --- | --- | --- | --- | --- | --- | --- | --- | --- | --- | --- | --- | --- | --- | --- |
| Treatment Group | Control | TAM | Bai | TAM+Bai | Control | TAM | Bai | TAM+Bai | Control | TAM | Bai | TAM+Bai | Control | TAM | Bai | TAM+Bai |
| Heart Markers | | | | | | | | | | | | | | | | |
| Left Ventricular Ejection Fraction (LVEF) | 0.74  ±0.03 | 0.70  ±0.02 | 0.70  ±0.03 | 0.69  ±0.01 | 0.72  ±0.02 | 0.73  ±0.01 | 0.72  ±0.01 | 0.69  ±0.07 | 0.69  ±0.02 | 0.70  ±0.03 | 0.69  ±0.01 | 0.71  ±0.02 | 0.73  ±0.01 | 0.71  ±0.02 | 0.73  ±0.01 | 0.72  ±0.04 |
| Systolic Blood Pressure (SBP, mmHg) | 115.2  ±5.1 | 121.0  ±6.5 | 124.3  ±5.3 | 116.3  ±3.9 | 119.4  ±11.5 | 119.1  ±4.6 | 119.0  ±1.3 | 114.6  ±9.5 | 120.7  ±0.2 | 116.7  ±5.6 | 117.4  ±4.7 | 114.0  ±1.4 | 117.1  ±0.8 | 113.4  ±4.5 | 120.0  ±4.0 | 121.1  ±2.4 |
| Diastolic Blood Pressure  (DBP, mmHg) | 70.2  ±4.0 | 73.2  ±3.3 | 69.7  ±2.7 | 73.7  ±1.6 | 70.7  ±2.3 | 67.1  ±0.6 | 71.6  ±0.6 | 69.7  ±2.1 | 73.7  ±5.9 | 72.7  ±5.6 | 68.9  ±1.8 | 69.8  ±3.2 | 73.7  ±3.4 | 73.5  ±4.6 | 78.4  ±0.8 | 74.2  ±3.5 |
| Stroke Volume  (SV, μL) | 40.0  ±6.0 | 36.9  ±1.4 | 34.2  ±2.8 | 31.5  ±3.4 | 39.1  ±2.6 | 44.0  ±6.1 | 37.8  ±2.8 | 37.2  ±8.4 | 36.5  ±3.5 | 41.5  ±8.8 | 43.6  ±2.0 | 41.3  ±8.6 | 46.4  ±6.2 | 43.9  ±2.2 | 50.3  ±6.8 | 45.5  ±10.8 |
| Heart Rate  (Bpm) | 442.3  ±10.0 | 439.0  ±13.6 | 452.0  ±24.1 | 438.7  ±9.8 | 445.3  ±17.6 | 437.3  ±8.3 | 431.8  ±22.7 | 442.7  ±25.3 | 461.3  ±13.0 | 443.0  ±11.6 | 438.7  ±11.9 | 427.7  ±13.5 | 451.7  ±11.3 | 442.0  ±13.5 | 433.7  ±7.6 | 430.7  ±6.8 |
| Liver Markers (Blood sample) | | | | | | | | | | | | | | | | |
| Alanine Transaminase (ALT, IU/L) | 37.0  ±1.7 | 34.7  ±4.6 | 35.2  ±0.6 | 36.4  ±0.7 | 32.9  ±2.8 | 33.5  ±2.2 | 35.4  ±4.1 | 33.5  ±2.4 | 35.5  ±2.3 | 40.5  ±6.9 | 36.5  ±1.6 | 38.6  ±2.5 | 39.4  ±2.4 | 37.0  ±1.0 | 36.9  ±5.1 | 35.8  ±3.2 |
| Aspartate Aminotransferase (AST, IU/L) | 88.9  ±7.4 | 85.3  ±4.3 | 84.2  ±8.0 | 85.7  ±6.6 | 89.3  ±7.1 | 82.2  ±9.2 | 80.3  ±4.4 | 78.0  ±11.3 | 86.9  ±6.2 | 89.7  ±7.0 | 83.4  ±8.8 | 88.2  ±3.9 | 80.5  ±4.5 | 77.2  ±7.8 | 72.6  ±7.1 | 84.3  ±6.3 |
| Kidney Markers (Blood sample) | | | | | | | | | | | | | | | | |
| Blood Urea Nitrogen (BUN, mM/L) | 10.1  ±0.4 | 9.6  ±0.9 | 9.3  ±0.9 | 9.2  ±0.1 | 9.9  ±1.0 | 10.8  ±1.1 | 10.9  ±1.1 | 11.5  ±2.9 | 9.7  ±0.8 | 9.7  ±0.4 | 9.9  ±1.0 | 10.1  ±0.6 | 10.0  ±0.8 | 9.5  ±0.4 | 10.0  ±0.3 | 10.0  ±0.5 |
| Serum Creatinine (Scr, μM/L) | 70.5  ±3.2 | 73.8  ±5.7 | 65.2  ±6.2 | 71.4  ±5.1 | 72.3  ±11.7 | 68.5  ±2.6 | 72.9  ±9.9 | 73.6  ±12.1 | 72.9  ±11.8 | 75.9  ±3.3 | 72.6  ±10.1 | 78.3  ±1.1 | 75.0  ±2.9 | 78.6  ±4.6 | 78.0  ±1.8 | 71.7  ±4.3 |
| Uric Acid  (UA, mM/L) | 46.9  ±5.7 | 48.3  ±4.0 | 47.9  ±3.2 | 42.2  ±6.5 | 49.7  ±4.4 | 45.5  ±5.4 | 47.7  ±6.3 | 45.9  ±8.6 | 40.8  ±5.0 | 38.0  ±5.0 | 42.7  ±5.9 | 39.6  ±4.5 | 42.6  ±1.9 | 42.6  ±4.3 | 41.2  ±4.3 | 44.6  ±2.8 |
